# Microbial degradation of jellyfish detritus promotes phytoplankton growth in coastal marine ecosystems

**DOI:** 10.1093/ismeco/ycag185

**Published:** 2026-07-01

**Authors:** Tinkara Tinta, Eduard Fadeev, Mauro Celussi, Cecilia Balestra, Katja Klun, Patricija Mozetič, Gerhard J Herndl

**Affiliations:** Marine Biology Station Piran, National Institute of Biology, 6330 Piran, Slovenia; Department of Functional and Evolutionary Ecology, University of Vienna, 1030 Vienna, Austria; Oceanography Division, National Institute of Oceanography and Applied Geophysics—OGS, 34151 Trieste, Italy; Oceanography Division, National Institute of Oceanography and Applied Geophysics—OGS, 34151 Trieste, Italy; Marine Biology Station Piran, National Institute of Biology, 6330 Piran, Slovenia; Marine Biology Station Piran, National Institute of Biology, 6330 Piran, Slovenia; Department of Functional and Evolutionary Ecology, University of Vienna, 1030 Vienna, Austria

**Keywords:** jellyfish blooms, organic matter degradation, microbial remineralization, phytoplankton dynamics, primary production, biogeochemical cycling, microscopy-based approach, metagenomics

## Abstract

Gelatinous zooplankton (hereinafter cnidarian *Medusozoa* and ctenophores or “jellyfish”) are widespread in marine ecosystems and can form blooms, releasing large amounts of labile, protein-rich organic matter (jelly-OM) upon decay. This material fuels intense bacterial activity, yet its ecological consequences remain poorly understood. We conducted a two-stage microcosm experiment simulating a bloom decay of the invasive ctenophore *Mnemiopsis leidyi* to examine microbial processing of jelly-OM and its effect on primary production (PP). In the first stage, over the course of 3 days, we observed jelly-OM stimulating rapid growth of opportunistic bacterial community. The community was dominated by *Pseudoalteromonadaceae*—key degraders of diverse jellyfish, which exhibited enhanced metabolism of amino acids, lipids, and carbohydrates and elevated extracellular enzymatic activities, including leucine aminopeptidase, lipase, chitinase, and alkaline phosphatase. These processes led to marked ammonium accumulation. In the second stage, exposure of a fresh microbial assemblage to residues from jelly-OM degradation resulted in a significant increase of PP and phytoplankton biomass over a period of five days. This was dominated by diatoms and was fueled by accumulated ammonium. Concurrently, the bacterial community shifted toward taxa typically associated with phytoplankton blooms. Together, these results, further supported by *in situ* observations, reveal a likely coupling between jellyfish decay and phytoplankton growth, suggesting that jellyfish blooms act as transient but powerful nutrient sources capable of triggering ecosystem shifts. As jellyfish are projected to thrive under future ocean conditions, our findings underscore the need to re-evaluate their role in biogeochemical cycles—particularly as overlooked drivers of phytoplankton dynamics.

## Introduction

Plankton bloom is a rapid and dense increase in the population of one or more species in a specific parcel of water. The presence of different bloom-forming organisms is increasing worldwide, especially in anthropogenically impacted coastal systems [1–3]. These organisms share specific biological and ecological traits that enable them to rapidly increase in abundance, including rapid reproduction, fast growth, resource efficiency, resistance to predation, adaptability to environmental change, and opportunism [4–6]. These features allow bloom-forming organisms to rapidly dominate marine ecosystems when conditions are favorable. They can act as both vital and potentially disruptive components, influencing trophic interactions, biogeochemical cycles and ecosystem resilience in response to climate change [4–6]. Hence, understanding the environmental triggers and consequences of bloom formation and decay is essential for predicting and effectively managing future changes in ocean ecosystems.

Phytoplankton blooms, among the most well studied examples of bloom-forming organisms in the ocean, are triggered by nutrients [7] and iron [8, 9] enrichment, light availability [10], temperature [11], and reduced grazing pressure [12]. Although phytoplankton comprise <1% of Earth’s photosynthetic biomass, they account for ~50% of global primary production (PP), fixing ~50 Pg C yr^−1^ and driving the export of ~10 Pg C yr^−1^ to the ocean interior via the biological carbon pump, highlighting their central role in marine ecosystems and global carbon cycling [13–15]. Increased phytoplankton blooms can enhance carbon sequestration and oxygen depletion and disrupt marine food webs [16].

While bloom-forming phytoplankton often receive significant attention [2, 17, 18], bloom-forming gelatinous zooplankton (hereinafter cnidarian *Medusozoa* and ctenophores or “jellyfish”) remain largely overlooked. Yet, jellyfish are widespread across diverse ecosystems, estimated to represent ~30% of total biovolume, corresponding to 9% of plankton carbon [19]. Due to their high adaptability and life history traits, some jellyfish populations can form large-scale blooms under favorable environmental conditions [20, 21]. Triggers of jellyfish blooms are less studied, but include food availability [6], eutrophication, habitat modification and climate change [22–24], with important socio-economic consequences, like disruption to marine food webs [25], alterations to biogeochemical cycles [26, 27] and effects on coastal economies [28]. The ability of jellyfish to thrive in changing oceanic environments suggests they may emerge as key players in future ocean scenarios, with potentially profound ecological and biogeochemical consequences [24]. Jellyfish are also increasingly recognized as significant contributors to the biological carbon pump, accounting for a substantial fraction of global particulate organic carbon export from the euphotic zone [29, 30].

The interactions between different (bloom-forming) organisms in the ocean are shaped by competition for resources, facilitation (i.e. an interaction in which the presence of one species alters the environment in a way that enhances growth, survival, or reproduction of a neighboring species), predation and trophic interactions, and environmental forcing [18, 31, 32]. Despite the growing recognition of phytoplankton and jellyfish blooms as features of marine ecosystems, the interactions between these two groups remain largely unexplored. While phytoplankton blooms provide the primary energy source for marine food webs, their potential role in fueling jellyfish blooms, either directly through consumption [33–38] or indirectly via trophic cascades [37, 39], is not well understood [27]. Similarly, the impact of jellyfish blooms on phytoplankton dynamics—whether via nutrient release [38, 40–42], through nutrient recycling [43–46], top–down control on grazers, or alterations in microbial interactions [37, 39]—remains poorly characterized [27, 41, 47]. Many jellyfish species exhibit boom-and-bust population dynamics, with important implications for ecosystems. Upon bust or decay of their blooms, nutrients stored in the biomass of these organisms become available to the surrounding system [43, 46]. The proteinaceous detrital material, characterized by low C: N ratio, supports a rapid increase in biomass of specific opportunistic bacteria, which efficiently re-mineralize complex organic jellyfish building blocks back to their inorganic constituents [43–47]. The “bust” or decay of jellyfish blooms could have a significant effect on PP through multiple biogeochemical pathways, influencing nutrient cycling, microbial communities, and oxygen dynamics. These interactions could stimulate or suppress PP, depending on environmental conditions and the organisms involved.

We propose that a key connection between bloom-forming jellyfish and phytoplankton may arise from the substantial release of inorganic nutrients, particularly ammonia and orthophosphate, during the bacterial degradation of jellyfish detritus [27, 43–46]. We hypothesize that these nutrients released during the decay phase of jellyfish blooms could play a significant role in fueling phytoplankton growth [27, 43–46]. In addition, end products of microbial remineralization of jellyfish detritus could alter phytoplankton communities’ composition by stimulating growth of species that thrive on recycled (e.g. dinoflagellates [48]) or high nutrient (e.g. diatoms [49]) conditions. Our hypothesis is supported by a limited set of studies exploring links between phytoplankton and jellyfish, revealing the co-occurrence of jellyfish outbreaks and increased phytoplankton growth [50–59]. These studies, however, do not provide evidence that decaying jellyfish blooms could be an important trigger of phytoplankton growth. To address our hypothesis, we simulated the scenario experienced by the coastal pelagic microbiome during a bloom of the invasive ctenophore *Mnemiopsis leidyi* in a two-stage microcosm experiment and following with high temporal resolution the abundance, metabolic activity and composition of the microbial community and specific microbial populations as well as the dynamics in the organic and inorganic nutrient pool. As proof-of-concepts, we confirmed our findings with *in situ* measurements.

## Materials and methods

### Experimental design

**Figure 1:**
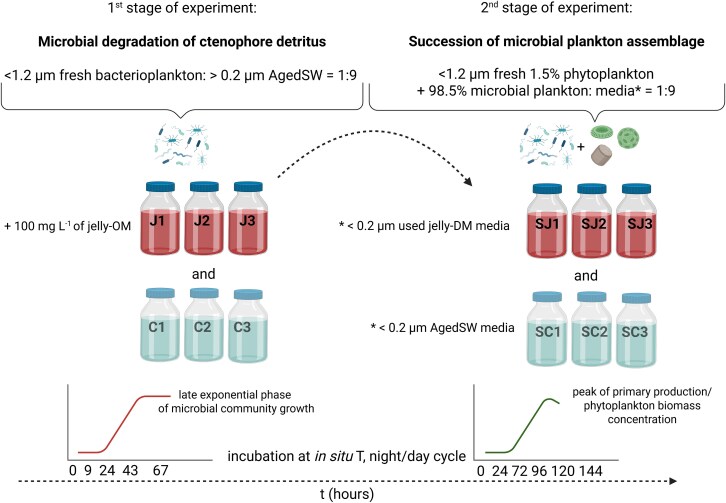
Experimental design.

In the first stage of the experiment, we incubated a coastal bacterioplankton assemblage with jellyfish-derived organic matter (hereinafter jelly-OM) at a concentration of 100 mg dry weight L^−1^, to approximate *in situ* concentration during bloom conditions, in the dark at *in situ* temperature. We described the rational for final concentration of jelly-OM as well as sampling and pre-processing of jellyfish biomass elsewhere in details [46, 60]; briefly, jelly-OM was obtained from *M. leidy* individuals sampled during the 2021 bloom in the northern Adriatic Sea, specimens were freeze-dried (−45°C for 7 days), pooled, and homogenized under sterile conditions. Treatments with bacterioplankton assemblage but without jelly-OM amendment served as controls. For each treatment we prepared biological triplicates (J1, J2, and J3 for jelly-OM and C1, C2, and C3 for control) by filling three 10 L borosilicate glass flasks with aged seawater (ASW) and freshly collected 1.2 μm pre-filtered coastal ambient seawater (serving as bacterioplankton inoculum) in a ratio of 9:1 (Fig. 1). All glassware used for experiments was 1 M HCl- and Milli-Q water-washed and combusted. Seawater for both ASW and the bacterial inoculum was collected at 5 m depth using a Niskin flask in the inner part of the Gulf of Trieste, northern Adriatic Sea (45°32′55.50″N, 13°33′2.52″E). Seawater used as ASW was aged in 20L Nalgen carboys for about one month at room temperature in the dark and pre-filtered through 0.2 μm prior the experiment. Seawater for the bacterioplankton inoculum was sampled and filtered on the day of the start of the experiment, when ambient seawater temperature was 23.7°C and salinity 35.5. We subsampled our experimental bottles at 0 h and after 9, 24, 43 and 67 h and enumerated prokaryotic abundance instantly via microscope after each subsampling to follow growth of prokaryotes. At each time point, subsamples were taken for microbial abundance, assessments of microbial metabolic activity at both community and single-cell levels and for dissolved organic matter (DOM) and inorganic nutrients from each of the experimental flasks, preserved and analyzed as described below. Once the bacterioplankton community reached the late exponential growth phase, bacterial biomass was collected for metagenomic analysis, as described below, and the 0.2 μm filtrate, containing end-products of microbial processing of jelly-OM was collected into three new 10 L borosilicate glass flasks (marked SJ1, SJ2, SJ3) (Fig. 1).

For stage two of the experiment, we sampled a fresh microbial plankton assemblage from 5 m depth using a Niskin flask. Phytoplankton was sampled by a vertical tow of a 20 μm mesh-size net from 14 m depth to the surface at the same location as for the first stage of our experiment. We enumerated prokaryotic abundance and examined one part of the phytoplankton net sample for its taxonomic composition using microscope. To prepare an inoculum that resembles the microbial plankton assemblage at the time of sampling we mixed 1.5% of the phytoplankton sample with 98.5% of 1.2 μm pre-filtered freshly collected coastal ambient seawater sample in 1 L and inoculated 9 L of jelly-OM processed media and ASW for the jelly-OM (SJ1, SJ2, SJ3) and control (SC1, SC2, SC3) treatments (Fig. 1). We followed the succession of phyto- and bacterioplankton communities by subsampling all bottles at 0 h and every 24 h until the end of the experiment at 144 h for microbial abundance, assessments of microbial metabolic activity at both community and single-cell levels and concentration of DOM and inorganic nutrients, as well as for chlorophyll *a* (Chl *a*) and PP. The phytoplankton abundance and taxonomy was analyzed using microscope at 0 h and at the end of the experiment. We terminated stage two of the experiment right after the peak of Chl *a* concentration was reached in the jelly-OM treatments (120 h after starting stage two of the experiment). Although oxygen was not directly measured in this experiment, previous highly reproducible incubations using a similar setup as in our first stage of experiment [43, 46] indicate that oxygen levels remained above ~80% saturation, likely due to regular mixing and aeration during high-frequency sampling.

### In situ *sampling*

At the end of our microcosm experiment we sampled the surface water within and outside the patch of a *M. leidyi* population at the same sampling station as for the microbial inoculum. Samples were collected with Niskin flask and processed and analyzed for prokaryotic and phytoplankton abundance and metabolic activity as well as for concentration of DOM and inorganic nutrients and metagenomic analysis.

### Prokaryotic abundance and production

Epifluorescence microscopy of DAPI-stained cells was used during the experiment to track microbial dynamics in near real time, as previously described [43]. The abundance of prokaryotes and phototrophic pico- and nanoplankton (Synechococcus, picoeukaryotes and nanoeukaryotes) was assessed using a FACSCanto II flow cytometer (Becton Dickinson BioSciences Inc., USA) equipped with an air-cooled argon laser at 488 nm and standard filters setup. The abundance of phototrophs was determined from unstained samples based on their natural fluorescence (phycoerythrin and Chl *a*; [61]) Samples for enumerating total prokaryotic abundance were diluted 10-fold with 0.2 μm filtered Tris-EDTA buffer (1×, Sigma Aldrich), stained with SYBR Green I (1× Final Concentration, Life Technologies), incubated in the dark at room temperature for 10 min according to Marie et al. [62] Methodological details are available in Supplementary Materials (File S1).

Bacterial carbon production (BCP) was determined using radiolabeled leucine (^3^H-Leu, Perkin Elmer, 20 nM final concentration) incorporation into newly synthesized proteins [63, 64]. The BCP was calculated as described by Simon and Azam [65] using a theoretical leucine-to-carbon conversion factor 3.1 kg C mol^−1^ Leu incorporated.

### Respiration and biomass production at the single-cell level

At 0 h and at the end of each experiment, 5 ml subsamples were spiked with Redox Sensor Green reagent (RSG, Thermo Fisher) at 1 μM final concentration and incubated for 30 min at *in situ* temperature in the dark to follow respiration of bacterial populations at the individual cell level. At the same time, 5 ml subsamples were spiked with the methionine analog L-homopropargylglycine (HPG, Thermo Fisher) reagent at 20 nM final concentration and incubated for 4 h at *in situ* temperature in the dark to follow production of bacterial populations at the individual cell level. Afterwards samples were fixed with formaldehyde (2% final concentration) and stored at −80°C until further analyzed as described previously [43]. To determine the abundance of respiring and biomass producing bacterial populations we applied the FISH method to the samples incubated with RSG and HPG, respectively, as previously described [43].

### Extracellular enzymatic activities

Extracellular enzymatic activities were assayed using fluorogenic substrate analogs [66] derived from 7-amino-4-methyl-coumarin (AMC) and 4-methyl-umbelliferone (MUF). Leucine aminopeptidase activity (AMA) was assayed as the hydrolysis rate of leucine-AMC. β-glucosidase, β-galactosidase, lipase (OLE), chitinase (CHIT) and alkaline phosphatase (APA) activities were assayed using MUF-β-D-glucoside, MUF-β-D-galactoside, MUF-oleate, MUF-N-acetyl-β-D-glucosaminide and MUF-phosphate, respectively (Sigma Aldrich). Hydrolysis was measured by incubating 0.3-ml sub-samples with 200 μM MUF-β-D-glucoside, MUF-β-D-galactoside, MUF-N-acetyl-β-D-glucosaminide, leucine-AMC, 100 μM MUF-oleate, and 50 μM MUF-phosphate for 1 h in the dark at the experimental temperature (at saturating concentrations [67]). The increase in fluorescence due to MUF and AMC hydrolyzed from the model substrate was measured using a Spark 20 M multimode microplate spectrofluorometer (TECAN) (MUF = 365-nm excitation and 455-nm emission; AMC = 380-nm excitation and 440-nm emission). All samples were run in five replicates. Triplicate calibration curves were performed using 0.2 μm filtered seawater and 5 μM MUF and AMC standard solutions.

### Phytoplankton abundance and production

Composition and abundance of phytoplankton were analyzed according to the Utermöhl method [68]. Methodological details are available in Supplementary Materials (File S1). Chl *a* concentrations as a proxy for phytoplankton biomass, corrected for phaeopigments, were determined fluorometrically in 90% acetone extracts on a Turner Trilogy fluorometer [69]. PP of phytoplankton was measured using the ^14^C method [70], as described in details in Supplementary Material (File S1). The assimilation of carbon was calculated as described by Gargas [71], considering a 5% isotope discrimination, the activity of the added ^14^C and the total CO_2_ concentration calculated from temperature, salinity and pH.

### Metagenomics

Bacterial biomass was collected onto 0.2 μm polyether-sulfone membrane filters (PES, PALL Inc.) by filtering 1.5 L of the (1.2 μm pre-filtered) bacterial inoculum, 300 ml from each of the triplicate control and jelly-OM treatments at the peak of bacterial abundance (43 h) and 1 L from each of the triplicate control and jelly-OM treatments at the end of stage one of our experiment (67 h). For stage two, microbial biomass was collected onto 0.2 μm PES filters (PALL Inc.) by filtering 2 L of the microbial plankton inoculum and 1 L from each of the triplicate control and jelly-OM treatments at the end of stage two of our experiment (144 h). From samples collected *in situ* within or outside the patch of *M. leidyi* bloom we filtered 1 L of seawater sample onto 0.2 μm PES filters (PALL Inc.). Filters were stored at −80°C until further processing.

For all samples, the DNA was extracted using DNeasy PowerWater kit according to the manufacturer’s instructions (Qiagen). Library construction and sequencing at Illumina NovaSeq (2 × 150bp) was outsourced to LGC Genomics (Germany). The entire metagenomic analysis was conducted with the Anvio software system [72] using snakemake [73] workflows implemented in the program “anvi-run-workflow”. Quality control of raw reads was carried out using illumina-utils [74]. Taxonomic classification of raw reads was performed using Kaiju [75] against the pre-built database “nr_euk” generated on 10 May 2023. The reads from each type of microcosm were pooled and co-assembled using MEGAHIT [76] and the reads mapping on the assemblies were carried out using Bowtie [77]. The reconstruction of metagenome assembled genomes (MAGs) was performed using MaxBin2 [78] metaBAT2 [79] and CONCOCT [80], and then refined by MetaWrap [81]. Using the program “anvi-estimate-metabolism” [82] on each MAG, we calculated the degree of completeness for a given KEGG module genomes using the annotation of genes with KEGG orthologs [82]. The program “anvi-compute-metabolic-enrichment” [83] was then used to determine whether a given metabolic module was enriched in certain microcosms.

### Chemical analyses

#### Dissolved organic carbon and nitrogen

Samples for dissolved organic carbon (DOC) and total dissolved nitrogen (TDN) were filtered onto combusted Whatman GF/F (~0.8 μm pore size) filters using acid-, Milli-Q water-rinsed and a combusted glass filtration system. Analyses were performed by a high temperature catalytic method using a Shimadzu TOC-L analyzer equipped with a total nitrogen unit [84]. The calibration for non-purgeable organic carbon was done with potassium phthalate and for TDN potassium nitrate was used. The results were validated with Surface-Sea Reference (SSR) water for DOC (CRM Program, Hansell Lab). DON was calculated by subtracting inorganic nitrogen nutrients (NO_2_^−^, NO_3_^−^, and NH_4_^+^) from TDN. The precision of the method expressed as RSD% was <2%.

#### Dissolved inorganic nutrients

Dissolved inorganic nitrogen (NH_4_^+^, NO_2_^−^, NO_3_^−^) and dissolved inorganic phosphorus (PO_4_^3−^) concentrations were determined spectrophotometrically by segmented flow analysis (QuAAtro, Seal Analytical) following standard methods [85]. The validation and accuracy of the results were checked with reference material (KANSO CO., LTD.) before and after sample analysis. The quality control is performed annually by participating in an intercalibration program (QUASIMEME Laboratory Performance Study). Dissolved silica was not measured due to limited sample volume and the prioritization of DOC analyses; all incubations were conducted in borosilicate bottles under identical conditions across treatments; thus silicate concentrations should be essentially the same in all the treatments.

#### Dissolved amino acid analysis

Samples for total dissolved amino acid analyses were filtered through combusted Whatman GF/F filters using acid-, Milli-Q water rinsed and combusted glass filtration systems. Approximately 2 ml of filtrate (two technical replicates) was collected in dark glass vial and stored at −20°C until analysis. Samples were analyzed for dissolved free amino acids (DFAA) and total dissolved hydrolysable amino acids (TDHAA) on a Shimadzu Nexera ×2 ultra-high-performance liquid chromatograph (UHPLC) with a fluorescence detector (RF-20A XS) as previously described [43]. The concentration of dissolved combined amino acids (DCAA) was calculated as the difference between TDHAA and DFAA.

### Statistical analysis

Statistical analyses were done in R v4.4.2 (R Core Team 2022) using RStudio v2024.09.1 (RStudio Team 2019). All figures were generated using the R package “ggplot2” v3.5.1 [86]. Scripts for data processing and statistical analyses can be accessed via Zenodo (https://www.zenodo.org/) under doi:https://doi.org/10.5281/zenodo.15780491.

### Data availability

The metagenomic raw sequences have been deposited in the European Nucleotide Archive (ENA) at EMBL-EBI under Project accession number PRJEB77875. Metadata for metagenomic samples deposited under accession PRJEB77875, including the associated experimental conditions is available in Table S1.

## Results and discussion

### Jellyfish-derived organic matter alters seawater stoichiometry and triggers a consistent metabolic response in the ambient microbiome

**Figure 2:**
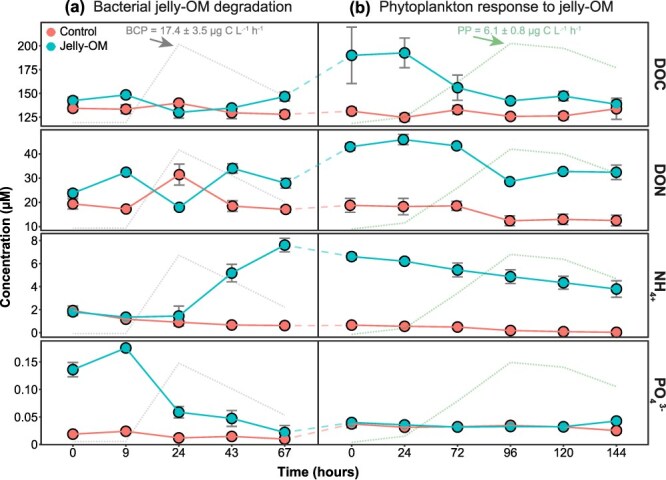
Dynamics of dissolved organic (DOC, DON) and inorganic nutrients (NH_4_^+^, PO_4_^3−^) and response of the microbial community–BCP (measured via ^3^H-Leu incorporation as a proxy for bacterial biomass synthesis; shown here only BCP for jelly-OM treatment) and PP (shown here only PP for jelly-OM treatment) measured via ^14^C-labeled bicarbonate as a proxy of photosynthetic carbon fixation during stage one (a) and stage two (b) of the incubation experiment in jelly-derived organic matter amended treatments (jelly-OM) and controls.

To create conditions resembling a realistic decaying jellyfish bloom, 100 mg of jelly-dry matter per liter (hereinafter jelly-OM) was added to ASW inoculated with ambient bacterioplankton assemblages [43, 48]. This resulted in an introduction of ~14 μmol L^−1^ DOC, ~12 μmol L^−1^ DON, ~1.3 μmol L^−1^ DFAA (including the sulfonic acid taurine which was with 579. ± 67 nmol L^−1^ the most abundant species in the pool of DFAA), ~1 μmol L^−1^ DCAA (within which glycine with 4.5 ± 1.5 nmol L^−1^ was most abundant), and ~ 1.5 μmol L^−1^ PO_4_^3−^ accumulating during the first 9 h of the first stage of our microcosm experiment (Fig. 2, Tables S2 and S5). This addition, closely resembling the results of our previous leaching experiments assessing the nutrient content of the jelly-OM pool [43, 48], altered the stoichiometry of the enclosed seawater DOM shifting the C:N ratio from 7.1 ± 1.3:1 to 4.7 ± 0.1:1. This nitrogen enrichment created more favorable conditions for bacterial growth, as lower C:N ratios are generally associated with increased substrate lability and nutritional quality [87]. As a result, bacterial communities responded with rapid growth and increased activity (μ = 0.104 ± 0.001 h^−1^; Table S3) between 9 and 24 h of the first stage of the experiment. This result aligns with our previous experiments, where we exposed a coastal microbiome to jelly-OM originated from *Aurelia* spp*.* and from ctenophore *M. leidyi*, resulting in bacterial growth rates of ~0.08 h^−1^ [43, 48]. These rates are comparable to those observed during short-lived pulses of organic matter in coastal systems, such as phytoplankton blooms (~0.1–0.2 h^−1^), but are notably higher than the average background rates typically reported for coastal environments (~0.008–0.08 h^−1^ [88]). Bacterial Carbon Production (BCP) peaked at 17.4 ± 3.5 μg C L^−1^ h^−1^, 24 h after jelly-OM amendment (Fig. 2). Bacterial abundance continued to increase, reaching ~8 × 10^6^ cells ml^−1^ by 67 h during the first stage of the experiment (Table S2, prokaryotic abundance, flow cytometry-based). Within this community, ~80% of cells were identified as Bacteria [EUB-positive], of which ~73% were respiring [RSG-positive] and ~ 51% were producing [HPG-positive] (Table S4). In contrast, in control treatments bacterial abundance remained constant at around 2 × 10^5^ cells ml^−1^ and BCP stayed low at 0.5 μg C L^−1^ h^−1^ until the end of the first stage of experiment. Within the control community, ~ 44% of cells were classified as Bacteria (EUB-positive), of which~48% were respiring [RSG-positive] and ~ 80% were biomass producing (HPG-positive) (Table S4).

During the first 24 h of the initial stage of the experiment, a substantial drawdown of DOC (18.3 ± 3.6 μmol L^−1^), TDN (13.6 ± 0.4 μmol L^−1^), DFAA (0.5 ± 0.1 μmol L^−1^, within which the entire pool of taurine and leucine was depleted), and PO₄^3−^ (0.12 ± 0.02 μmol L^−1^) were observed, likely supporting the bacterial growth recorded during this period (Fig. 2, Tables S2, S3, and  S5). The heterotrophic bacterial carbon demand (BCD) was assumed to be only fueled by DOC and was decreasing on average by 14.7 ± 2.9 μg C L^−1^ h^−1^ during exponential growth phase (from 9-24 h of the experiment, Tables S2 and S3) in jelly-OM amended bottles. The BCD represents the total carbon consumed for the synthesis of new bacterial biomass (bacterial production, BP) and the amount of organic carbon respired (bacterial respiration, BR). We estimated BP also from the increase in bacterial biomass over time, calculated from the differences in bacterial abundance, assuming C-content of 19.8 fg C cell^−1^ [89]. In this way, we estimated a BR of 9.6 ± 4.2 μg C L^−1^ h^−1^ during exponential growth of the bacterial community exposed to jelly-OM enrichment (Table S3). From the BCD and BP, we estimated a bacterial growth efficiency (BGE = BP/BCD) of 40% ± 20% in jelly-OM treatments (Table S3). This is in line with our previous experiments with jelly-OM of the ctenophore *M. leidyi* (18%–27% [46]), and lower than estimated from experiments exposing a coastal microbiome to jelly-OM of *Aurelia aurita* s.l. (~30%–80% [43, 44]). These BGE values are comparable to those measured during phytoplankton blooms (~20%–60%) or, in general, in nutrient-rich zones (~10%–50%), but altogether higher than those reported for coastal waters (~5%–30%) [88, 90, 91]. To summarize, we reproducibly showed that organic material of different bloom-forming jellyfish species supports rapid growth of the coastal microbiota, which not only respire carbon stored in jellyfish biomass, but also use it to build up microbial biomass (reaching on average 45.4 ± 11.6 μg C L^−1^, Table S3).

After the first 24 h, when most of the labile jelly-OM had been depleted by the bacterial community and a decline in bacterial productivity had been observed, concentrations of DOC and DON increased by ~20 μmol L^−1^ and 30 μmol L^−1^, respectively (Fig. 2, Table S2). The observed increase in DOC and DON was likely fueled by bacterial processing of complex compounds originating from the particulate fraction of jelly-OM, which we did not measure due to technical limitations. By the end of the first stage of our experiment, we observed a substantial accumulation of ammonium (NH₄^+^) in the jelly-OM treatments, reaching concentrations of ~7 μmol L^−1^, which is well above the concentrations measured in the ambient seawater during the same season in the region (Fig. 2, Table S2). This finding is consistent with previous studies [27, 43, 46] and is most likely attributable to ammonification—the microbially mediated process by which ammonium is generated during the mineralization of low molecular weight dissolved organic compounds containing amine or amide functional groups.

### Jellyfish-derived organic matter induces distinct shifts in microbial community composition and metabolism

**Figure 3:**
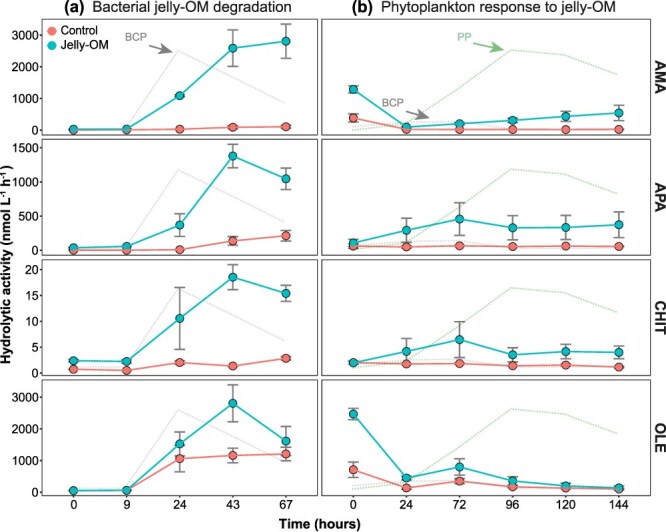
Extracellular enzymatic activities measured using fluorogenic substrate analogs derived from 7-amino-4-methyl coumarin (AMC) and 4-methly umbelliferone MUF. Leucine aminopeptidase (AMA), alkaline phosphatase (APA), chitinase (CHIT) and lipase (OLE) during stage one (a) and stage two (b) of the incubation experiment in jelly-derived organic matter amended treatments (jelly-OM) and controls. The development of BCP and PP is indicted by the faint dotted line.

The degradation of the labile jelly-OM was characterized by enhanced leucine aminopeptidase (AMA), lipase (OLE), chitinase (CHIT), and alkaline phosphatase (APA) activity (Fig. 3, Fig. S1). Following the peak in BCP at 24 h in the first experiment, extracellular enzymatic activities (EEA) in the jelly-OM treatment were substantially elevated compared to the control (Fig. 3). Specifically, leucine AMA was 28-fold higher (and coincided with a drawdown of all the leucine from the dissolved combined amino acid pool, Table S5), APA was 10-fold higher, CHIT was 14-fold higher, and OLE was 2.4-fold higher than in the control (Fig. 3). This profile of EEA differs from the repertoire typically associated with bacterial degradation of phytoplankton detritus, where elevated activities of various glycoside hydrolases are commonly observed [67, 92, 93]. The differences in EEA profiles observed in bacterial communities degrading jelly-OM versus phytoplankton-OM reflect the contrasting biochemical composition of the protein-rich jelly-OM versus carbohydrate-rich phytoplankton-OM. These compositional differences likely drive the selection of distinct microbial assemblages, with potentially important implications for the structure and function of coastal microbiomes. Specifically, jelly-OM may favor microbial taxa equipped with the genomic capacity to degrade proteins, whereas phytoplankton-OM supports the growth of carbohydrate-degrading microorganisms.

**Figure 4:**
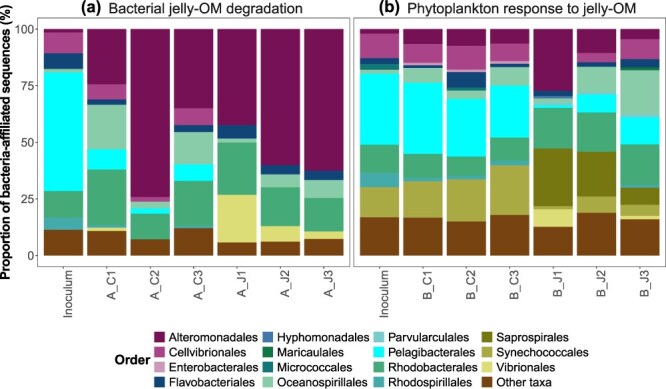
Relative abundance of bacteria-affiliated sequences at the end of the stage one (a—after 67 h of the jelly-OM bacterial degradation experiment), and at the end of the stage two (b—after 144 h of the phytoplankton response experiment), of the incubation experiment.

**Figure 5:**
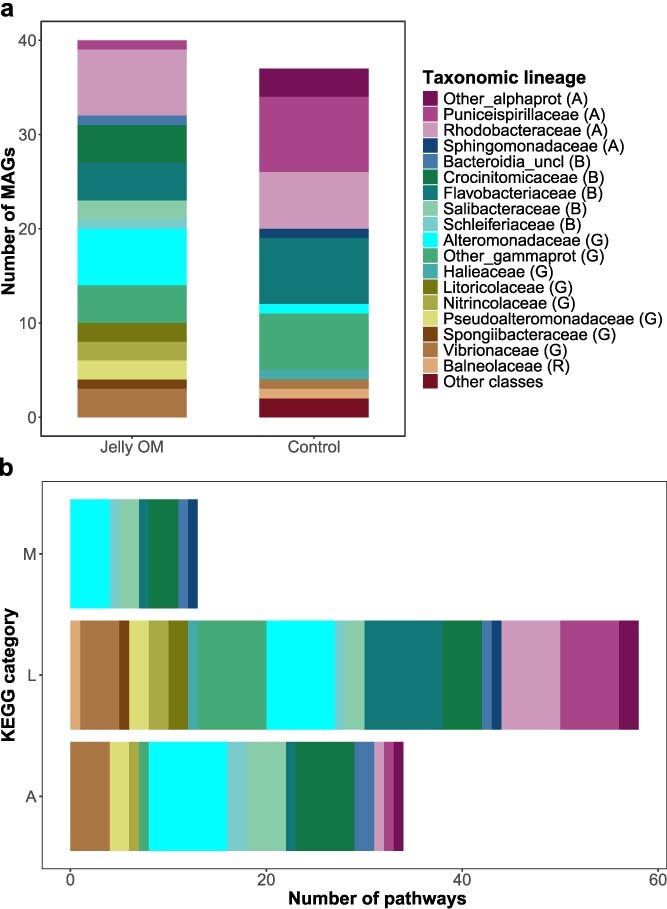
Differences in metabolic capabilities between jelly-OM and control treatments. (a) Number of reconstructed metagenome-assembled genomes (MAGs) according to taxonomic affiliation. (b) Number of significantly enriched metabolic pathways in the MAGs corresponding to the KEGG categories: “A”—amino acid metabolism, “L”—lipid metabolism, and “M”—metabolism of cofactors and vitamins. The colors are the same in the two panels, letters in parentheses represent the taxonomic class of the lineage: “A”—Alphaproteobacteria, “B”—Bacteroidia, “G”—Gammaproteobacteria, and “R”—Rhodothermia.

Metagenomic sequencing revealed that enhanced bacterial growth in the jelly-OM microcosms was primarily associated with opportunistic lineages, such as *Alteromonadales* and *Vibrionales* (both class Gammaproteobacteria), representing 43%–63% and 3%–21% of bacterial sequences, respectively, compared to <8% in the control microcosms (Fig. 4a). Using the metagenomes, we reconstructed a total of 40 metagenome-assembled genomes (MAGs) from the jelly-OM microcosms, half of which were affiliated to gammaproteobacterial lineages (among those were two MAGs of *Pseudoalteromonadaceae,* three MAGs of *Vibrionaceae*), and additional eight MAGs were affiliated with alphaproteobacterial lineages (7 of which affiliated with *Rhodobacteraceae*; Fig. 5). In contrast, in the control microcosms, out of 37 MAGs, only 10 were affiliated with gammaproteobacterial lineages (among which 6 MAGs were affiliated with *Pseudomonadales* and 1 with *Vibrionaceae*), and 18 MAGs were affiliated with alphaproteobacterial lineages (of which 8 MAGs affiliated with *Puniceispirillaceae* and 6 MAGs affiliated with *Rhodobacteraceae*). Metabolic enrichment analysis between the MAGs in both microcosms revealed significantly enriched (adjusted q-value <0.1) metabolic pathways related to lipid metabolism (e.g. beta-oxidation), amino acid metabolism (e.g. histidine degradation) and metabolism of cofactors and vitamins (e.g. NAD biosynthesis pathways) in the bacterial communities of the jelly-OM microcosms (Fig. 5). No significantly enriched pathways were observed in the control microcosms. The similarity to our previous jelly-OM degradation experiments is striking—regardless of the year or the specific group of jellyfish, jelly-OM consistently triggers a structural shift in the ambient microbiome, with largely the same bacterial lineages driving its remineralization [27, 43, 44, 46].

### Residues of bacterial degradation of jellyfish-derived organic matter promote phytoplankton growth

**Figure 6:**
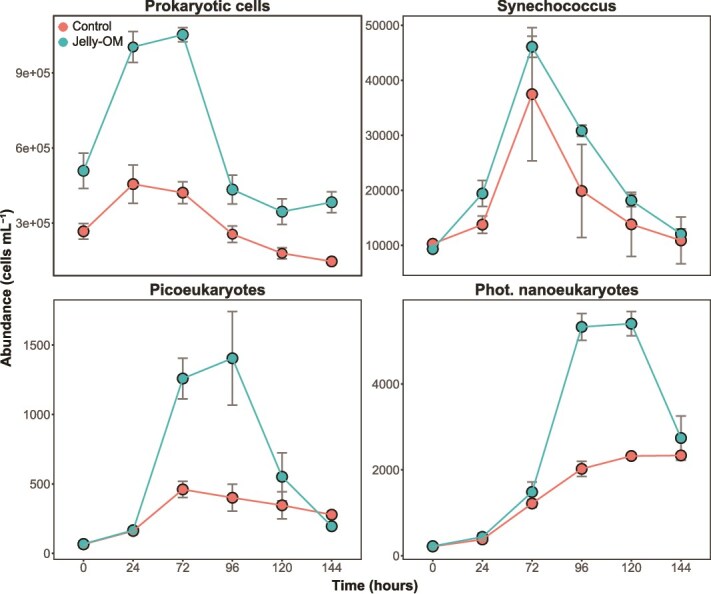
Cell abundance dynamics of microbial communities in response to jelly-OM residues, as revealed by flow cytometry.

In stage two, the 0.2 μm pre-filtered medium from stage one of the experiment initially supported the growth of the freshly collected ambient microbial plankton community by carrying over residues of degraded jelly-OM (Figs 1 and 2). We recorded an increase in bacterial abundance from 5.1 ± 1.1 × 10^5^ cell ml^−1^ at 0 h to 1.1 ± 0.5 × 10^6^ cell ml^−1^ after 72 h, which coincided with a peak in BCP (1.8 ± 0.7 μg C L^−1^ h^−1^). Based on metagenomic sequences, the bacterial community exposed to jelly-OM residues was dominated by *Saprospirales* (class Saprospiria; 9%–26% of bacterial sequences), *Rhodobacterales* (ca. 18% of bacterial sequences) and *Alteromonadales* (6%–28% of bacterial sequences; Fig. 4), taxa commonly reported to be associated with phytoplankton blooms [94, 95]. After 96 h, in the microcosms amended with jelly-OM residues a drawdown of ~48 μmol L^−1^ DOC and ~ 14 μM μmol L^−1^ DON was recorded (Fig. 2b, Table S2). By contrast, in the unamended control microcosms, we observed BCP levels that were more than an order of magnitude lower, with bacterial communities reaching twofold lower cell abundances (Fig. 6, Table S3). These communities were dominated by *Pelagibacterales* (class Alphaproteobacteria) and *Synechococcales* (class Cyanophyceae), which comprised 23%–32% and 16%–22% of bacterial sequences, respectively.

Although we did not detect significantly enriched metabolic pathways nor enhanced extracellular enzymatic activity in the bacterial communities exposed to jelly-OM residues compared to the control (Fig. 3, Fig. S1), the observed taxonomic difference is likely linked to further bacterial processing of residual DOM (e.g. DOC, DON) derived from jelly-OM in the second stage of the experiment.

**Figure 7:**
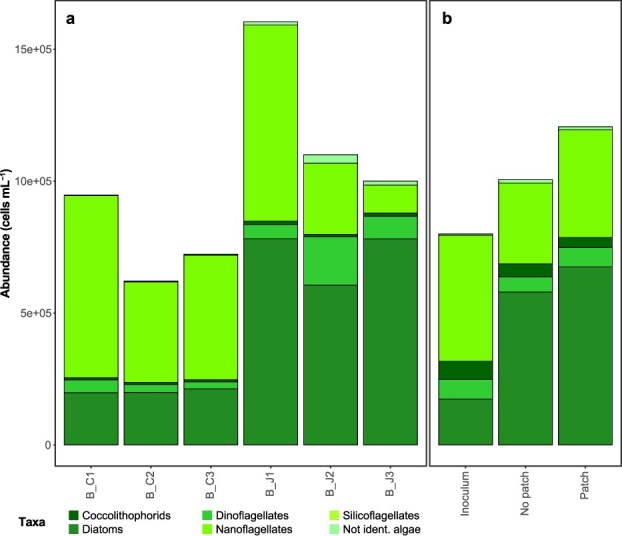
Phytoplankton community composition as revealed by microscopy counts. (a) In microcosm experiments in response to jelly-OM and (b) in natural marine microbial communities (inoculum for the second stage of microcosm experiment, outside (No patch) and inside (patch) *M. leidyi* patch).

We observed an increase in the phytoplankton biomass and production in treatments amended with jelly-OM residues after 24 h, with the primary production (PP) reaching 6.1 ± 1.4 μg C L^−1^ h^−1^ after 96 h and the biomass reaching 1.2 ± 0.3 μg Chl *a* L^−1^ after 120 h (Fig. 2b). This represents an ~30-fold increase in Chl *a* concentration compared to the initial inoculum (< 0.1 μg L^−1^), reaching or even exceeding values otherwise observed *in situ* during seasonal phytoplankton peaks in the region [96, 97]. The enclosed phytoplankton community in the unamended controls remained relatively stable, exhibiting a minor peak in PP after 96 h with 1.87 ± 0.08 μg C L^−1^ h^−1^ that supported an increase in biomass up to 0.30 ± 0.03 μg Chl *a* L^−1^ (Table S2). The observed phytoplankton growth on jelly-OM residues was likely supported by ammonium carried over from the first stage of the experiment. This is evidenced by a continuous decrease in ammonium concentrations—from 6.6 ± 0.5 μM at the start (0 h) to 3.8 ± 1.2 μM after 144 h (Fig. 2, Table S2), whereas nitrate and nitrite concentrations remained stable at 0.50 ± 0.02 μM and 0.04 ± 0.02 μM, respectively (Table S2). Although silica was not measured, identical borosilicate bottles across treatments were used, indicating that observed differences in phytoplankton dynamics were driven by jelly-OM addition. As phytoplankton biomass and production increased, we observed a sharp decline in bacterial abundance in the treatments containing jelly-OM residues—from 1.05 ± 0.49 × 10^6^ to 4.3 ± 1.0 × 10^5^ cells ml^−1^ within just 24 h (Fig. 6). The observed dynamic is in line with several studies providing evidence that phytoplankton preferentially utilizes ammonium as source of nitrogen [98] due to lower assimilation energy costs [99] and high affinity ammonium transporters [100, 101]. It has been shown that under certain conditions phytoplankton can outcompete bacteria for this nitrogen source [102] or when regenerated ammonia becomes available, for example, through ammonification during bacterial remineralization of protein-rich jelly-derived organic matter (jelly-OM) [95, 103]. Residuals of the degraded jelly-OM likely induced a structural change within the phytoplankton community, from nanoflagellates to diatoms, which represented 49%–78% of phytoplankton cells taxa at the end of the experiment (after 144 h), compared to 22% in the initial inoculum (at 0 h), as determined by microscopy (Fig. 7). This community dynamic corresponds with previous studies showing that diatoms can outcompete other phytoplankton groups, such as photosynthetic or mixotrophic flagellates, for ammonium as source of nitrogen [104–106]. Additionally, flow cytometry-based analysis revealed that, while *Synechococcus* exhibited similar dynamics to heterotrophic bacteria, phototrophic picoeukaryotes (0.2–2 μm in size, with representatives of mostly small chlorophytes) sharply increased in abundance after the first 24 h of the second stage of experiment, reaching a peak of abundance between 72–96 h following the peak of heterotrophic bacteria, and dramatically dropped in abundance right afterwards. In contrast, phototrophic nanoeukaryotes (2–20 μm in size, with typical representatives such as haptophytes, cryptophytes, chrysophytes and other nanoflagellates as well as small diatoms) started to increase in abundance only after the decline of heterotrophic bacteria, *Synechococcus* and picoeukaryotes, reaching an abundance peak after 120 h, coinciding with a peak in Chl *a* concentration, followed by sharp decline at the end of the experiment. These dynamics revealed potentially complex interactions between different groups of primary producers and heterotrophic bacteria that remain to be explored.

We also conducted *in situ* measurements within a *M. leidyi* patch during the peak of its bloom in fall 2021 in the Gulf of Trieste, northern Adriatic Sea. We measured slightly higher Chl *a*, ammonium, and orthophosphate concentrations within the *M. leidyi* patch (0.6 ± 0.08 μg Chl *a* L^−1^, 0.2 ± 0.2 μM, and 0.026 ± 0.019 μM, respectively) as compared to the ambient waters (0.36 ± 0.11 μg Chl *a* L^−1^, 0.03 ± 0.03 μM and 0.018 ± 0.004 μM, respectively) (Table S2). However, PP and BCP, as well as metagenomic and microscopy-based analyses of phytoplankton and bacterial communities, reveal no significant differences within the *M. leidyi* patch, compared to ambient waters (Table S2, Fig. 7b, Fig. S2). These preliminary results suggest the need for more *in situ* measurements in the future, with a higher temporal and spatial resolution. Efforts must also be made to overcome the logistical challenges posed by the patchy and unpredictable nature of jellyfish blooms, as well as the current lack of tools for remotely tracking their development and population dynamics.

In conclusion, our study reveals a previously overlooked link between decaying jellyfish blooms and enhanced phytoplankton growth. In the aftermath, decaying jellyfish biomass may induce major perturbations to ecosystems by stimulating growth of specific opportunistic bacteria, which rapidly remineralize jelly-OM, releasing inorganic nutrients and thereby, stimulating primary production. Since jellyfish are expected to thrive under future ocean conditions—such as warming, deoxygenation, and increased exploitation of marine resources, our study highlights the importance of re-evaluating the role of jellyfish blooms in marine biogeochemical cycles and considering them as potential, yet previously unaccounted, drivers of phytoplankton dynamics.

## Supplementary Material

Supplementary_material_ycag185

## Data Availability

All data needed to evaluate the conclusions of the article are present in the article and/or the Supplementary Material. The metagenomic raw sequences have been deposited in the European Nucleotide Archive (ENA) at EMBL-EBI under Project accession number PRJEB77875. Metadata for metagenomic samples deposited under accession PRJEB77875, including the associated experimental conditions is available in Table S1.
