# Supplementary material for: Microbial degradation of jellyfish detritus promotes phytoplankton growth in coastal marine ecosystems
Source: ISME Commun. 2026 Jul 1;6(1):ycag185. doi: 10.1093/ismeco/ycag185 (PMC13452463; doi:10.1093/ismeco/ycag185)
Supplement: Supplementary file 1 [file supplementary_material_ycag185.zip › Table S2.pdf]

Table S2 - Concentration of dissolved organic and inorganic nutrients, including chlorophyll a and primary production, throughout two stage microcosm experiment in jelly-OM amended bottles and in control treatments with no jelly-OM addition.

DOC - dissolved organic carbon [μmol L^{-1}],
TDN - total dissolved nitrogen [μmol L^{-1}],
NH_4 - ammonium [μmol L^{-1}],
NO_2 - nitrite [μmol L^{-1}],
NO_3 - nitrate [μmol L^{-1}],
DIN - dissolved innorganic nitrogen [μmol L^{-1}],
DON - dissolved organic nitrogen [μmol L^{-1}],
TDHAA - total hydrolysable dissolved amino acids [μmol L^{-1}],
DFAA - total dissolved free amino acids [μmol L^{-1}],
DCAA - total dissolved combined amino acids [μmol L^{-1}],
PO_4^{3} - phosphate [μmol L^{-1}],
Chl. a - chlorophyll *a* [μg L^{-1}],
PP - Primary production [μg C L^{-1} h^{-1}]
N - Bacterial abudance [cells mL^{-1}]
BCP - Bacterial carbon production based on ^{3}H-Leu incorporation [μg C L^{-1} h^{-1}]
The values represent a mean and standard error of three biological replicates.

Experiment	Treatment	Time	DOC	TDN	NH_4^+	NO_2^-	NO_3^-	DIN	DON	TDHAA	DCAA	DFAA	PO_4^{3-}	Chl. a	PP	Cell abund.	BCP
Bac. deg.	Jelly-OM	0	142.42±3.69	25.66±1.63	1.81±0.31	0.01±0	0.26±0.03	2.08±0.34	23.58±1.32	2396.77±13.64	1174.02±18.31	1283.84±51.53	0.14±0.01	NaN±NA	NaN±NA	133±10	0.01±0
Bac. deg.	Jelly-OM	9	148.42±2.38	33.3±1.07	1.35±0.09	0.02±0	0.16±0.01	1.52±0.1	31.77±0.98	2745.33±35.9	1754.46±191.11	1419.29±55.6	0.18±0	NaN±NA	NaN±NA	147±11	0.05±0.01
Bac. deg.	Jelly-OM	24	130.06±6.07	19.7±1.1	1.45±0.86	0.01±0	0.16±0.01	1.62±0.88	18.08±0.88	2929.1±579.82	2263.73±664.25	920.45±58.21	0.06±0.01	NaN±NA	NaN±NA	253±18	17.39±3.52
Bac. deg.	Jelly-OM	43	134.67±0.63	38.62±2.55	5.18±0.76	0.01±0	0.18±0.03	5.37±0.77	33.25±1.81	3278.11±177.12	3154.48±197.63	524.57±75.59	0.05±0.01	NaN±NA	NaN±NA	540±161	11.72±0.54
Bac. deg.	Jelly-OM	67	146.67±4.65	35.25±2.3	7.61±0.59	0.01±0	0.16±0.01	7.78±0.6	27.47±1.91	2634.02±NA	2540.94±NA	292.66±78.48	0.02±0.01	NaN±NA	NaN±NA	584±209	5.88±0.31
Bac. deg.	Control	0	134.31±3.26	21.58±2.42	1.92±0.4	0.01±0	0.24±0.05	2.18±0.44	19.41±2.01	1695.78±544.94	1642.34±544.75	75.95±15.12	0.02±0	NaN±NA	NaN±NA	114±8	0.02±0
Bac. deg.	Control	9	133.39±4.62	18.77±0.81	1.17±0.08	0.01±0	0.17±0.01	1.36±0.09	17.41±0.72	1213.25±230.82	1155.56±225.68	73.12±16.64	0.02±0	NaN±NA	NaN±NA	128±6	0.05±0
Bac. deg.	Control	24	139.78±1.84	31.91±4.15	0.91±0.05	0.01±0	0.16±0.01	1.08±0.06	30.83±4.09	1373.18±15.65	1316.91±21.6	86.94±26.43	0.01±0	NaN±NA	NaN±NA	2271±579	0.54±0.14
Bac. deg.	Control	43	129.67±6.19	19.39±2.21	0.67±0.11	0.01±0	0.16±0.02	0.84±0.13	18.55±2.09	1428.93±308.66	1381.54±307.45	65.71±10.5	0.01±0	NaN±NA	NaN±NA	6186±144	0.43±0.13
Bac. deg.	Control	67	127.97±4.41	18.05±1.18	0.62±0.09	0.01±0	0.17±0.02	0.8±0.11	17.25±1.07	1507.33±567.44	1463.91±560.6	76.62±18.61	0.01±0	NaN±NA	NaN±NA	8268±599	0.2±0.04
Phyt. res.	Jelly-OM	0	189.97±29.65	48.84±1.51	6.61±0.29	0.03±0	0.53±0.08	7.17±0.35	41.67±1.35	2528.62±304.58	2349.66±349.86	233.44±71.72	0.04±0	0.04±0.01	0.49±0.05	267±31	1.24±0.85
Phyt. res.	Jelly-OM	24	192.56±15.61	51.22±1.83	6.2±0.2	0.04±0	0.48±0.05	6.72±0.2	44.5±2.02	2085.75±62.37	1907.04±112.05	253.62±97.87	0.04±0	0.14±0.01	0.92±0.06	456±76	1.74±0.49
Phyt. res.	Jelly-OM	72	156.03±13.27	48.03±0.72	5.44±0.6	0.04±0	0.51±0.03	6±0.57	42.03±0.63	2169.28±183.54	2042.51±175.81	133.48±29.75	0.03±0	0.5±0.03	3.38±0.21	421±44	1.84±0.39
Phyt. res.	Jelly-OM	96	142.17±1.63	33.54±0.98	4.86±0.6	0.06±0.02	0.51±0.04	5.43±0.55	28.1±0.43	2251.74±337.22	2094.39±366.34	163.22±47.24	0.03±0	1.07±0.1	6.1±0.82	256±33	0.96±0.29
Phyt. res.	Jelly-OM	120	147.19±4.84	36.95±0.8	4.34±0.56	0.06±0.02	0.49±0.03	4.89±0.54	32.06±0.68	2028.27±235.95	1843.11±287.87	209.28±63.91	0.03±0	1.21±0.2	5.76±0.46	179±22	0.93±0.3
Phyt. res.	Jelly-OM	144	138.58±4.18	36.06±2.55	3.79±0.71	0.07±0.02	0.48±0.02	4.34±0.69	31.72±2.83	2027.46±76.74	1804.37±75.16	235.62±53.9	0.04±0	0.94±0.13	4.4±0.72	147±10	1.02±0.37
Phyt. res.	Control	0	131.31±3.7	19.83±2.67	0.66±0.03	0.02±0	0.3±0.06	0.99±0.07	18.84±2.72	1150.12±107.01	1051.38±99.16	100.9±9.54	0.04±0.01	0.05±0	0.36±0.05	509±71	0.23±0.02
Phyt. res.	Control	24	124.69±4.14	19.17±3.21	0.56±0.01	0.02±0	0.22±0.03	0.8±0.02	18.36±3.19	1076.17±143.56	966.68±138.18	111.12±7.19	0.03±0	0.1±0	0.76±0.06	1004±62	0.23±0.01
Phyt. res.	Control	72	132.83±4.27	19.37±1.74	0.49±0.06	0.02±0	0.21±0.02	0.72±0.04	18.65±1.78	1225.51±18.15	1117.28±22.74	109.81±4.1	0.03±0	0.21±0.02	1.43±0.08	1053±28	0.1±0
Phyt. res.	Control	96	125.78±2.6	13.24±1.9	0.19±0.05	0.02±0	0.21±0.02	0.43±0.03	12.81±1.93	978.84±546.22	924.96±519.11	185.55±98.46	0.03±0	0.3±0.02	1.89±0.05	434±58	0.07±0
Phyt. res.	Control	120	126.36±3.84	13.7±2.03	0.09±0.03	0.02±0	0.19±0.02	0.31±0.01	13.39±2.04	1391.45±184.26	1293.1±190.54	99.07±6.08	0.03±0	0.3±0.01	1.8±0.13	346±51	0.05±0
Phyt. res.	Control	144	133.86±11.07	13.15±2.11	0.04±0.02	0.02±0	0.18±0.03	0.23±0.04	12.92±2.08	1170.54±114.76	1080.97±111.5	91.51±4.04	0.03±0	0.32±0.02	1.62±0.1	383±42	0.05±0
In situ	Patch	-	128.56±1.47	14.58±0.35	0.22±0.12	0.02±0.01	0.93±0.1	1.17±0.22	13.41±0.45	1853.2±194.32	1649.51±136.71	209.54±76	0.03±0.01	0.6±0.04	3.2±0.1		0.3±0
In situ	No. Patch	-	157.58±15.63	18.64±1.86	0.04±0.02	0.02±0	0.84±0.02	0.9±0.03	17.74±1.85	1544.58±103.98	1369.93±92.16	182.84±31.21	0.02±0	0.36±0.07	3.2±0.11		0.28±0.01
