# Supplementary material for: Microbial degradation of jellyfish detritus promotes phytoplankton growth in coastal marine ecosystems
Source: ISME Commun. 2026 Jul 1;6(1):ycag185. doi: 10.1093/ismeco/ycag185 (PMC13452463; doi:10.1093/ismeco/ycag185)
Supplement: Supplementary file 1 [file supplementary_material_ycag185.zip › Table S3.pdf]

Table S3 - Bacterial community growth parameters during exponential growth phase in the first stage of our microcosm experiment in jelly-OM amended bottles and in control treatments with no jelly-OM amendment

The values represent a mean and standard deviation of three biological replicates.
BCD - bacterial carbon demand [μg C L^{-1} h^{-1}].
BB - [μg C L^{-1}]
BP - bacterial productivity [μg C L^{-1} h^{-1}].
BR - bacterial respiration [μg C L^{-1} h^{-1}].
BGE - bacterial growth efficiency.
μ - cell doubling time [h^{-1}]

Treatment	BCD	BB	BP	BR	BGE	μ
Control	1.8±0.51	10.8±3.22	0.17±0.09	1.63±0.56	0.12±0.07	0.01±0
Jelly-OM	14.69±2.95	45.42±11.58	4.76±1.28	9.93±4.17	0.4±0.2	0.1±0
