# Supplementary material for: Microbial degradation of jellyfish detritus promotes phytoplankton growth in coastal marine ecosystems
Source: ISME Commun. 2026 Jul 1;6(1):ycag185. doi: 10.1093/ismeco/ycag185 (PMC13452463; doi:10.1093/ismeco/ycag185)
Supplement: Supplementary file 1 [file supplementary_material_ycag185.zip › Table S4.pdf]

Table S4 - Abundance of microbial cells (DAPI), bacterial cells (D+F (DAPI positive cells hybridized with EUB I, II and III probe)), respiring and HPG incorporating microbes (D+H (DAPI and RSG/HPG positive cells)) and respiring and HPG incorporating bacteria (D+F+H (DAPI and RSG/HPG positive cells hybridized with EUB I, II and III probe) at the start (0h) and the end (67, 144h) of each of the stage (Bac. deg, Phyt.res) of our microcosm experiment in jelly-OM amended bottles and in control treatments with no jelly-OM amendment.

The values represent a mean and standard deviation of three biological replicates.
RSG - Redox Sensor Green reagent
HPG - methionine analog L-homopropargylglycine

D+F = DAPI+FISH signal
D+H = DAPI + HPG/RSG signal
D+F+H = DAPI+FISH+HPG/RSG signal

Probe	Experiment	Treatment	Time	DAPI		D+F		D+H		D+F+H	
				cells/mL	SD	cells/mL	SD	cells/mL	SD	cells/mL	SD
RSG	Bac. deg.	Jelly-OM	0	1,43E+05	6,08E+04	5,85E+04	9,12E+03	2,62E+04	2,12E+04	2,17E+04	2,45E+04
RSG	Bac. deg.	Jelly-OM	67	6,35E+06	6,13E+05	5,62E+06	8,20E+05	4,57E+06	8,53E+05	4,10E+06	9,40E+05
RSG	Bac. deg.	Control	0	1,66E+05	6,14E+03	3,73E+04	1,83E+04	2,19E+04	6,71E+03	1,06E+04	5,24E+03
RSG	Bac. deg.	Control	67	5,01E+05	2,80E+05	2,75E+05	1,87E+05	1,63E+05	1,45E+05	1,34E+05	1,11E+05
RSG	Phyt. res.	Jelly-OM	S 0	2,98E+05	7,47E+04	1,68E+05	7,81E+04	4,49E+04	2,21E+04	4,29E+04	2,46E+04
RSG	Phyt. res.	Jelly-OM	S 144	7,44E+05	1,74E+05	4,26E+05	2,14E+05	2,04E+05	1,02E+05	1,88E+05	9,89E+04
RSG	Phyt. res.	Control	S 0	4,05E+05	7,46E+04	2,09E+05	1,31E+05	8,45E+04	7,64E+04	8,07E+04	7,30E+04
RSG	Phyt. res.	Control	S 144	1,49E+05	1,17E+05	2,32E+04	2,43E+04	1,46E+04	1,37E+04	1,05E+05	1,58E+05
HPG	Bac. deg.	Jelly-OM	0	2,66E+05	1,68E+05	1,31E+05	1,10E+05	4,09E+04	2,91E+04	2,61E+04	7,49E+03
HPG	Bac. deg.	Jelly-OM	67	3,90E+06	1,04E+06	2,83E+06	7,40E+05	1,82E+06	4,20E+05	1,45E+06	1,53E+05
HPG	Bac. deg.	Control	0	8,78E+04	4,92E+04	2,88E+04	4,28E+03	2,65E+04	1,18E+04	2,04E+04	5,35E+03
HPG	Bac. deg.	Control	67	4,56E+05	2,15E+05	1,59E+05	6,87E+04	2,73E+05	9,20E+04	1,32E+05	3,41E+04
HPG	Phyt. res.	Jelly-OM	S 0	2,95E+05	2,54E+05	2,12E+05	2,42E+05	2,12E+05	2,22E+05	1,78E+05	2,29E+05
HPG	Phyt. res.	Jelly-OM	S 144	3,52E+05	3,96E+04	1,36E+05	5,91E+04	1,70E+05	6,45E+04	1,01E+05	7,75E+04
HPG	Phyt. res.	Control	S 0	2,30E+05	7,72E+04	1,01E+05	5,03E+04	1,05E+05	6,66E+04	6,00E+04	3,50E+04
HPG	Phyt. res.	Control	S 144	7,61E+04	1,32E+05	3,39E+04	5,88E+04	9,63E+03	1,67E+04	9,63E+03	1,67E+04
