# Supplementary material for: Microbial degradation of jellyfish detritus promotes phytoplankton growth in coastal marine ecosystems
Source: ISME Commun. 2026 Jul 1;6(1):ycag185. doi: 10.1093/ismeco/ycag185 (PMC13452463; doi:10.1093/ismeco/ycag185)
Supplement: Supplementary file 1 [file supplementary_material_ycag185.zip › Table S5.pdf]

Table S5 - Concentration of dissolved free amino acids, dissolved combined amino acids and total hydrolysable dissolved amino acids throughout two stage microcosm experiment in jelly-OM amended bottles and in control treatments with no jelly-OM addition.

DFAA - Dissolved Free Amino Acids [nM]
DCAA - dissolved combined amino acids [nM]
TDHAA - total hydrolysable dissolved amino acids [nM]

The values represent a mean and standard error of three biological replicates.

Experiment	Treatment	Time	Fraction	Asp acid	Glu acid	Asn	Ser	Gln	Gly	His/Thr	Arg	Ala	Tau	GABA	Tyr	Val	Met	Trp	Phe	Iso	Leu	Lys	Asx	Glx	His
Bac. deg.	Control	T0	DFAA	18.86±0.35	5.89±1.11	0.62±0.13	3.43±0.67	0.36±0.18	5.55±0.71	0.78±0.12	0.91±0.06	2.33±0.21	1.55±0.49	0±0	0.15±0.15	20.32±15.38	0.47±0.11	1.12±0.4	1.32±1.32	4.05±2.61	0.87±0.05	7.36±1.64			
Bac. deg.	Control	T1	DFAA	18.33±1.6	5.43±1.15	0.6±0.13	4.95±2.9	0.43±0.09	5.54±2.17	1.08±0.4	0.89±0.05	2.87±1.22	1.31±0.45	0±0	0.44±0.28	15.4±7.16	0.31±0.16	0.81±0.41	4.82±2.91	3.34±2.08	0.91±0.22	5.66±0.7			
Bac. deg.	Control	T2	DFAA	18.36±1.24	5.37±1.16	0.64±0.1	5±3.07	0.42±0.08	3.83±1.97	0.99±0.5	0.41±0.09	2.56±1.16	0.44±0.18	0±0	0.25±0.25	35.83±26.1	0.43±0.19	0.11±0.06	5.05±3.19	2.63±1.37	0.59±0.16	4.04±1.86			
Bac. deg.	Control	T3	DFAA	17.66±0.52	7.87±0.39	0.61±0.04	3.83±1.49	0.85±0.05	3.19±0.73	0.67±0.23	0.34±0.06	1.93±0.47	0.08±0.01	0±0	0.23±0.23	16.62±11.87	0.31±0.18	0.11±0.06	3±1.53	1.83±0.92	0.66±0.31	5.93±0.78			
Bac. deg.	Control	T4	DFAA	17.03±0.31	9.19±0.96	0.71±0.09	1.44±0.2	1.23±0.25	1.79±0.11	0.5±0.04	0.43±0.17	1.18±0.08	0.22±0.07	0±0	0.12±0.12	31.96±21.33	0.5±0.15	0.89±0.61	1.03±1.03	2.82±1.46	0.76±0.08	4.81±0.72			
Bac. deg.	Jelly-OM	T0	DFAA	68.43±5.63	114.29±5.5	18.55±1.68	28.12±3.32	68.09±4.34	29.5±1.81	33.48±2.28	93.54±6.39	15.41±0.78	578.93±38.57	0.93±0.47	16.96±1.14	79.4±23.52	11.07±0.12	5.01±0.48	13.6±1.16	20.01±1.01	24.43±1.63	64.1±3.76			
Bac. deg.	Jelly-OM	T1	DFAA	71.99±5.31	122.24±1.87	21.19±0.14	26.22±0.43	72.21±0.72	27.76±0.29	35.58±0.58	67.38±33.69	47.54±29.45	440.21±180.46	6.17±5.47	266.87±248.43	69.22±32.85	9.31±2.07	5.59±0.21	15.3±1.02	21.29±0.61	25.69±0.43	67.53±0.92			
Bac. deg.	Jelly-OM	T2	DFAA	65.98±0.67	57.12±8.19	4.42±2.29	8.09±3.66	5.88±3.69	11.08±4.7	5.71±2.96	3.73±0.58	0±0	510.35±86.82	1.4±0.02	4.33±1.45	110.33±15.91	0.42±0.42	2.52±1.66	2.18±2.18	111.82±42.25	2.73±0.74	12.38±2.09			
Bac. deg.	Jelly-OM	T3	DFAA	49.75±0.75	19.53±1.86	1.36±0.29	3.23±0.39	2.51±0.33	3.53±0.18	0.98±0.16	1.67±0.2	2.19±0.29	1.28±0.04	0.18±0.18	1.67±0.37	173.76±7.13	0.78±0.78	1.53±0.05	0±0	250.56±72.81	0.69±0.34	9.36±0.35			
Bac. deg.	Jelly-OM	T4	DFAA	44.77±1.14	23.05±1.74	0.33±0.11	1.62±0.26	2.29±0.37	1.89±0.14	0.59±0.09	0.93±0.15	1.67±0.45	0.99±0.12	0±0	3.1±0.91	136.75±39.51	0.3±0.16	2.43±0.48	6.31±6.31	57.04±42.23	0.66±0.05	7.94±1.25			
Bac. deg.	Control	T0	TDHAA				164.03±68.91		311.06±108.09		48.45±21.18	126.36±20.9	21.01±3.84	18.82±1.08	18.13±12.73	389.71±195.11			33.95±18.95		48.34±27.89	92.19±39.84	235.28±30.8	140.66±52.84	47.8±10.99
Bac. deg.	Control	T1	TDHAA				89.7±5.75		184.72±11.26		26.93±2.77	99.92±1.68	16.66±1.28	18.05±2.47	8.74±4.39	365.42±184.02			22±11.09		16.83±2.24	54.93±18.39	195.37±7.86	76.22±0.64	37.76±2.33
Bac. deg.	Control	T2	TDHAA				52±26.12		117.87±58.98		17.46±9.01	64.39±32.2	10.86±5.49	11.86±6.04	6.89±3.61	363.86±182.62			20.14±10.75		9.69±5	45.67±22.95	137.63±44.3	49.28±24.68	24.23±12.11
Bac. deg.	Control	T3	TDHAA				117.39±7.89		226.81±27.81		38.2±5.4	112.52±6.29	14.46±1.37	18.71±0.36	11.22±5.08	380.6±192.77			28.67±14.34		28.71±6.33	64.9±21.09	274.07±59.43	72.09±38.19	40.59±3.91
Bac. deg.	Control	T4	TDHAA				77.15±41		157.44±81.65		27.12±14.62	71.58±35.8	10.96±5.49	13.23±6.74	14.84±7.6	259.4±259.4			28.32±16.25		23.79±13.17	65.21±34.45	165.22±65.65	74.81±40.19	29.23±14.65
Bac. deg.	Jelly-OM	T0	TDHAA				75.27±37.64		237.77±119.6		141.58±70.8	95.07±47.59	436.23±218.12	6.66±3.58	15.62±8.12	0±0			0±0		39.4±19.72	51.47±25.79	223.72±91.08	231.34±115.75	57.95±29
Bac. deg.	Jelly-OM	T1	TDHAA				158.02±29.32		507.42±68.3		230.83±3.38	188.61±17.71	667.07±7.86	21.04±3.49	37.79±4.48	0±0			0±0		82.01±10.16	95.73±9.5	376.47±18.21	274.27±137.21	106.06±5.23
Bac. deg.	Jelly-OM	T2	TDHAA				167.05±18.55		705.68±174.45		132.08±17.4	244.46±65.07	561.28±85.77	17.34±5.31	29.26±6.51	0±0			1.65±1.65		84.46±28.29	71.61±17.99	438.08±116.78	366.89±213.4	109.26±28.71
Bac. deg.	Jelly-OM	T3	TDHAA				154.32±77.79		694.41±349.37		81.82±40.91	247.03±123.83	27.6±15.39	9.83±4.92	30.47±15.28	24.02±24.02			0±0		84.24±42.22	56.16±28.12	448.35±200.78	230.6±227.49	114.5±57.6
Bac. deg.	Jelly-OM	T4	TDHAA				60.98±60.98		260.82±260.82		27.6±27.33	91.11±91.11	13.57±13.57	6.87±6.87	8.49±8.49	0±0			0±0		25.65±25.65	16.63±16.63	191.25±134.37	179.53±168.89	41.37±40.66
Bac. deg.	Control	T0	DCAA				160.59±69.23		305.51±108.16		47.54±21.18	124.03±20.96	19.46±3.37	18.82±1.08	17.98±12.77	386.25±193.27			32.63±17.87		47.48±27.87	84.83±41.04	215.8±30.58	134.4±51.75	47.01±11.08
Bac. deg.	Control	T1	DCAA				84.75±6.61		179.18±9.52		26.04±2.72	97.05±1.07	15.36±1.44	18.05±2.47	8.42±4.21	357.52±180.96			20.53±10.62		15.92±2.08	49.27±19.07	176.44±9.06	70.36±1.25	36.68±2.16
Bac. deg.	Control	T2	DCAA				47.85±23.93		114.79±57.4		17.17±8.85	62.36±31.23	10.53±5.31	11.86±6.04	6.64±3.57	356.91±178.73			18.74±10.61		9.31±4.78	44.02±22.26	118.62±44.09	45.81±22.91	23.46±11.74
Bac. deg.	Control	T3	DCAA				113.56±6.81		223.62±27.49		37.86±5.46	110.58±5.93	14.38±1.38	18.71±0.36	10.99±4.98	377.42±191.29			25.66±12.83		28.05±6.2	58.97±21.87	255.8±59.91	66.01±35.3	39.92±3.75
Bac. deg.	Control	T4	DCAA				76.09±40.46		156.17±81.04		26.94±14.55	70.81±35.41	10.83±5.43	13.23±6.74	14.72±7.52	257.73±257.73			27.29±15.37		23.26±12.9	62.47±33.13	147.48±65.63	67.44±36.39	28.9±14.49
Bac. deg.	Jelly-OM	T0	DCAA				54.35±27.21		216.9±109.22		74.98±37.49	84.56±42.38	24.78±13.7	6.14±3.54	3.55±2.79	0±0			0±0		22.05±11.03	6.28±3.26	151.52±75.84	103.23±51.83	34.35±17.18
Bac. deg.	Jelly-OM	T1	DCAA				131.8±29.45		479.67±68.54		163.45±31.53	141.06±40.26	226.86±176.85	14.87±2.14	14.03±8.11	0±0			0±0		56.32±9.99	28.2±8.88	283.29±19.08	144.43±72.54	70.48±5.8
Bac. deg.	Jelly-OM	T2	DCAA				158.96±21.28		694.59±179.13		128.35±17.93	244.46±65.07	50.93±5.05	15.93±5.3	24.93±7.96	0±0			1.65±1.65		81.73±28.99	59.24±19.85	367.68±118.52	331.7±201.25	103.55±31.43
Bac. deg.	Jelly-OM	T3	DCAA				151.92±76.58		692.08±348.23		80.79±40.4	245.62±123.15	26.78±15.02	9.83±4.92	29.46±14.81	0±0			0±0		83.87±42.06	50.15±25.12	398.43±199.63	220.19±220.19	113.86±57.3
Bac. deg.	Jelly-OM	T4	DCAA				60.31±60.31		260.1±260.1		27.01±27.01	90.25±90.25	13.16±13.16	6.87±6.87	6.86±6.86	0±0			0±0		25.43±25.43	13.77±13.77	146.14±134.23	164.43±164.43	40.93±40.51
Phyt. res.	Control	T0	DFAA	18.81±0.83	6.98±0.9	0.45±0.23	2.77±0.83	0.72±0.72	4.19±0.38	0.88±0.11	0.5±0.25	2.38±0.32	2.57±0.35	0±0	0±0	49.79±3.21	0±0	0±0	0±0	2.16±1.49	0±0	8.7±2.88			
Phyt. res.	Control	T1	DFAA	18.87±0.29	6.37±0.86	0.88±0.52	2.97±0.61	0.72±0.72	3.12±0.45	0.25±0.25	0.09±0.04	0.95±0.51	0.64±0.2	0±0	0±0	53.48±2.46	0±0	0±0	4.17±2.09	1.62±1.62	0±0	16.99±2.65			
Phyt. res.	Control	T2	DFAA	19.36±0.61	7.07±0.33	0.55±0.24	1.76±0.16	1.56±0.35	1.88±0.94	0.2±0.2	0.29±0.19	1.43±0.18	1.2±0.27	0±0	0±0	58.27±2.56	0±0	0±0	2.08±2.08	2.62±1.32	0±0	11.53±1.32			
Phyt. res.	Control	T3	DFAA	19.79±0.95	7.58±0.5	0.68±0.11	3.15±1.1	1.92±0.3	4.89±1.31	0.75±0.38	1.34±0.6	1.72±1.07	1.5±0.56	0±0	0±0	131.23±98.95	0±0	0±0	1.27±1.27	1.17±1.17	1.28±1.28	7.27±3.49			
Phyt. res.	Control	T4	DFAA	18.85±0.75	6.31±0.3	0.21±0.13	2.92±0.3	0.47±0.27	4.06±0.4	0.59±0.3	0.64±0.14	2.09±0.07	1.52±0.18	0.15±0.15	0±0	41.85±5.23	0±0	0±0	2.07±1.74	0.72±0.72	0±0	16.63±1.01			
Phyt. res.	Control	T5	DFAA	19.15±0.4	5.36±0.45	0.67±0.11	3.51±0.66	0.74±0.38	4.1±0.23	0.79±0.4	1.11±0.64	1.89±0.48	1.93±0.35	0.81±0.31	0±0	31.15±0.18	0±0	0±0	0.35±0.35	1.94±0.98	0.21±0.21	17.81±3.11			
Phyt. res.	Jelly-OM	T0	DFAA	40.52±1.4	24±1.13	0.38±0.21	2.16±0.28	2.48±0.62	2.74±0.09	0.45±0.23	1.14±0.05	2.35±0.21	1.55±0.17	0.66±0.66	3.54±1.22	93.11±46.65	0.11±0.06	1.21±0.66	0±0	53.16±33.54	0.29±0.22	3.59±1.81			
Phyt. res.	Jelly-OM	T1	DFAA	36.3±2.32	18.51±2.25	0.96±0.39	3.3±1.36	2.19±0.94	2.71±0.61	0.63±0.38	0.79±0.12	1.83±0.37	1.12±0.15	0±0	3.58±0.91	95.59±47.79	0.06±0.06	1.07±0.06	0±0	73.78±52.92	0.04±0.04	11.15±5.9			
Phyt. res.	Jelly-OM	T2	DFAA	26.56±0.53	18.12±3.52	0.76±0.16	1.47±0.29	5.57±1.34	3.37±0.66	0.59±0.03	0.91±0.13	1.6±0.17	1.52±0.14	0±0	3.74±0.87	46.02±34.25	0.09±0.09	1.36±0.45	9.98±5.23	4.52±4.51	0±0	7.29±1.86			
Phyt. res.	Jelly-OM	T3	DFAA	25.7±0.04	16.05±1.35	1.66±0.52	2.42±0.65	9.46±4.34	5.02±0.45	0.87±0.15	1.57±0.31	2.7±0.26	2.21±0.36	0±0	5.08±0.45	68.86±51.29	0.13±0.07	1.21±0.61	8.49±4.55	4.32±2.83	0.53±0.26	8.16±1.97			
Phyt. res.	Jelly-OM	T4	DFAA	27.48±0.19	23.01±1.23	2.53±0.2	3.22±0.04	24.5±4.27	12.42±3.31	1.16±0.02	2.79±0.22	6±1.03	5.59±0.06	1.24±0.61	4.44±1.01	70.55±53.63	0.1±0.1	0.72±0.72	5.39±5.39	7.54±3.69	1.06±0.08	9.53±2.76			
Phyt. res.	Jelly-OM	T5	DFAA	17.86±8.93	15.61±8.05	1.65±0.22	2.69±0.3	15.58±1.02	20.56±8.37	1.12±0.16	2.77±0.1	6.67±1.37	5.77±1.16	1.63±0.14	4.06±1	113.03±28.36	0.15±0.08	0.27±0.17	0±0	11.65±0.78	1.14±0.08	13.4±5.13			
Phyt. res.	Control	T0	TDHAA				80.62±15.13		185.51±8.61		21.35±0.83	105.01±7.7	17.86±2.21	15.71±4.02	9.68±0.45	323.87±55.51			24.01±4.53		19.15±2.79	74.32±8.66	155.36±12.59	79.19±6.02	38.49±2.97
Phyt. res.	Control	T1	TDHAA				86.91±13.44		196.27±11.13		24.24±1.02	94.84±13.06	16.03±1.19	12.74±6.57	9.37±1.82	249.17±34.67			21.67±1.92		22.17±2.36	89.48±5.47	148.74±37.82	68.11±14.78	36.43±4.86
Phyt. res.	Control	T2	TDHAA				61.8±31.3		126.3±63.17		8.94±7.55	36.87±36.87	42.71±34.9	13.92±6.97	7.29±3.87	240.17±120.22			15.94±7.99		12.67±6.34	53.21±26.83	117.57±53.25	55.81±27.91	27.89±13.96
Phyt. res.	Control	T3	TDHAA				82.51±46.93		150.36±78.9		18.12±10.33	79.44±41.29	11.01±5.72	13.6±6.8	10.41±5.82	238.45±119.41			67.73±57.56		60.4±52.33	39.67±39.67	109.4±59.71	69.34±38.3	28.41±15.2
Phyt. res.	Control	T4	TDHAA				128.32±39.26		211.52±21.63		27.33±1.97	117.27±15.6	23.44±5.83	23.2±2.18	14.7±1.79	361.83±47.23			23.79±2.1		23.59±4.9	109.59±14.04	186.4±19.75	94.33±8.48	46.15±7.27
Phyt. res.	Control	T5	TDHAA				96.41±5.89		207.62±4.75		21.13±0.21	109.76±3.77	18.08±1.05	17.87±1.38	13.08±1.27	289.51±73.72			16.02±8.14		23.16±1.7	93.4±11.13	144.2±14.58	80.24±7.09	40.07±2.02
Phyt. res.	Jelly-OM	T0	TDHAA				160.5±40.59		393.39±68.87		68.92±15.22	172.61±18.75	26.3±1.38	22.05±2.12	31.2±6.88	809.96±36.35			48.67±4.03		55.38±18	154.86±18.2	289.97±39.4	221.99±34.51	72.81±10.6
Phyt. res.	Jelly-OM	T1	TDHAA				123.76±8.6		318.33±0.55		44.27±5.58	157.85±2.11	33.15±3.59	21.33±2.74	17.43±1.71	702.42±71.05			45.62±2.16		29.6±2.16	108.21±1.31	260.26±3.6	158.42±4.54	65.11±0.82
Phyt. res.	Jelly-OM	T2	TDHAA				119.32±5.47		328.67±20.72		41.83±5.41	190.62±21.21	16.2±8.23	22.61±3.98	21.23±3.16	736.33±93.45			46.03±5.27		37.78±3.81	120.78±11.62	262.99±15.37	176.54±14.01	48.33±24.28
Phyt. res.	Jelly-OM	T3	TDHAA				154.66±22.74		282.73±116.8		48±9.41	165.12±10.53	30.82±1.43	22.83±1.19	25.66±4.29	767.49±85.02			53.31±4.33		46.53±10.8	142.99±19.9	262.52±16	198.65±30.36	50.42±25.25
Phyt. res.	Jelly-OM	T4	TDHAA				129.57±10.67		332.67±23.18		49.24±4.94	159.13±10.84	26.91±0.62	24.84±3.42	22.86±3.44	597.58±66.16			45.56±3.48		40.54±4.93	121.87±9.77	251.23±22.61	158.72±79.49	67.55±6.87
Phyt. res.	Jelly-OM	T5	TDHAA				122.64±8.86		316.65±12.76		39.16±5.38	155.88±7.2	35.34±4.1	21.57±0.53	15.14±5.19	658.84±51.28			42.3±3.28		34.52±1.66	115.57±10.66	250.06±2.05	175.97±7.67	43.83±22.03
Phyt. res.	Control	T0	DCAA				77.85±15.43		181.32±8.32		20.85±1.07	102.63±7.46	15.3±1.85	15.71±4.02	9.68±0.45	274.07±52.6			24.01±4.53		19.15±2.79	65.62±8.02	136.11±12.68	71.49±4.44	37.6±3.01
Phyt. res.	Control	T1	DCAA				83.94±13.94		193.15±11.51		24.15±0.98	93.89±12.59	15.39±0.99	12.74±6.57	9.37±1.82	195.69±34.05			17.5±0.69		22.17±2.36	72.49±4.81	128.99±38.13	61.02±13.91	36.18±4.7
Phyt. res.	Control	T2	DCAA				60.64±30.75		125.41±62.71		8.87±7.48	36.33±36.33	42.09±34.72	13.92±6.97	7.29±3.87	201.44±100.77			13.86±7.32		12.67±6.34	44.76±22.76	99.78±50.59	49.92±24.98	27.89±13.96
Phyt. res.	Control	T3	DCAA				80.96±45.89		147.94±77.65		17.62±10.16	77.72±40.3	9.64±5.04	13.6±6.8	10.41±5.82	216.92±108.71			66.46±56.29		60.4±52.33	35.61±35.61	96.1±53.27	63.53±35.72	28.06±14.92
Phyt. res.	Control	T4	DCAA				125.41±39.44		207.47±22.02		26.7±1.86	115.17±15.65	21.91±5.82	23.05±2.31	14.7±1.79	319.98±50.71			21.71±1.89		23.59±4.9	92.97±14.74	167.35±20.35	87.54±8.39	45.56±7.57
Phyt. res.	Control	T5	DCAA				92.9±6.54		203.52±4.97		20.02±0.78	107.87±4.18	16.15±1.38	17.06±1.3	13.08±1.27	258.35±73.55			15.68±8.03		22.94±1.87	75.59±9.18	124.38±14.5	74.14±7.07	39.27±1.8
Phyt. res.	Jelly-OM	T0	DCAA				158.34±40.43		390.64±68.79		67.78±15.27	170.27±18.74	24.75±1.21	21.39±1.57	27.66±7.93	716.85±77.89			48.67±4.03		55.1±17.77	151.27±16.63	249.08±40.26	195.51±35.9	72.35±10.38
Phyt. res.	Jelly-OM	T1	DCAA				120.46±7.29		315.61±0.66		43.48±5.68	156.01±1.75	32.03±3.56	21.33±2.74	13.85±2.01	606.83±110.6			45.62±2.16		29.56±2.2	97.05±5.72	223±5.46	137.71±1.97	64.48±1.19
Phyt. res.	Jelly-OM	T2	DCAA				117.85±5.18		325.3±20.31		40.92±5.31	189.02±21.31	15.21±7.72	22.61±3.98	17.49±3.96	690.31±84.29			36.05±2.11		37.78±3.81	113.49±13.22	235.66±15.03	152.85±10.22	47.95±24.09
Phyt. res.	Jelly-OM	T3	DCAA				152.24±22.12		277.71±116.47		46.43±9.13	162.42±10.29	28.61±1.34	22.83±1.19	21.78±4.84	698.63±128.76			44.82±3.82		46±10.67	134.83±20.82	235.17±15.47	173.14±24.99	49.76±24.91
Phyt. res.	Jelly-OM	T4	DCAA				126.35±10.64		320.25±25.07		46.45±4.75	153.13±11.72	21.32±0.63	23.6±3.76	18.42±4.45	527.03±118.26			40.17±8.87		39.47±4.88	112.35±12.03	221.22±22.83	126.96±63.48	66.39±6.89
Phyt. res.	Jelly-OM	T5	DCAA				119.94±8.7		296.09±4.7		36.4±5.47	149.21±5.82	29.57±4.29	19.94±0.66	11.08±5.09	545.81±58.99			42.3±3.28		33.38±1.63	102.17±8.93	230.54±8.79	144.78±0.39	43.17±21.7
Env. samp.	Env. samp.	M0	DFAA	25.13±2.72	12.73±1.56	1.24±0.15	8.6±2.35	1.97±0.23	12.24±2.3	2.21±0.42	2.09±0.39	5.53±0.98	6.15±0.5	0.49±0.49	1.56±0.08	62.31±10.71	0±0	3.06±0.37	2.66±0.72	5.13±1.14	1.11±0.21	28.63±8.94			
Env. samp.	Env. samp.	ML	DFAA	25.69±2.97	14.9±3.93	1.61±0.79	17.25±9.34	2.18±1.1	17.83±8.27	3.51±1.43	3.58±1.69	8.99±4.27	7.88±3.34	0.92±0.92	2.15±1.1	48.62±12.98	0±0	1.2±1.2	3.1±1.77	4.65±2.96	2.24±1.26	43.24±25.06			
Env. samp.	Env. samp.	M0	TDHAA				121.28±5.94		229.91±11.05		34.48±2.83	137.01±6.47	24.64±2.02	18.36±0.74	14.83±1.2	382.93±33.97			29.5±2.23		32.39±2.93	128.06±19.07	222.79±11.96	114.15±7.36	54.25±2.55
Env. samp.	Env. samp.	ML	TDHAA				166.2±21.96		297.04±37.54		37.12±4.1	179.85±10.8	29.78±5.39	97.51±72.76	19.27±1.73	356.81±66.66			38.28±3.47		45.63±5.08	187.38±36.35	194.17±28.51	141.66±21.42	62.49±4.36
Env. samp.	Env. samp.	M0	DCAA				112.67±6.81		217.67±11.02		32.38±2.63	131.48±6.2	18.49±1.99	17.87±1.07	13.27±1.25	320.62±27.87			26.83±2.44		31.28±3.02	99.43±11.79	196.43±12.26	99.46±6.28	52.04±2.76
Env. samp.	Env. samp.	ML	DCAA				148.95±15.15		279.21±31.86		33.53±5.09	170.86±8.22	21.9±2.67	96.58±73.24	17.12±1.23	308.2±54.8			35.19±2.5		43.39±4.37	144.14±12.52	166.87±24.8	124.59±17.88	58.99±3.28
