# Supplementary material for: Microbial degradation of jellyfish detritus promotes phytoplankton growth in coastal marine ecosystems
Source: ISME Commun. 2026 Jul 1;6(1):ycag185. doi: 10.1093/ismeco/ycag185 (PMC13452463; doi:10.1093/ismeco/ycag185)
Supplement: Supplementary file 1 [file supplementary_material_ycag185.zip › Table_S1.pdf]

Experiment	Treatment	Time	Run_accession	Sample_accession	Experiment_accession	fastq_ftp	bam_ftp
Bac. deg.	-	0	ERR13395277	SAMEA115854230	ERX12765939	ftp.sra.ebi.ac.uk/vol1/fastq/ERR133/077/ERR13395277/ERR13395277_1.fast	ftp.sra.ebi.ac.uk/vol1/err/ERR133/077/ERR13395277/ERR13395277.bam
Bac. deg.	Control	43	ERR13395281	SAMEA115854234	ERX12765943	ftp.sra.ebi.ac.uk/vol1/fastq/ERR133/081/ERR13395281/ERR13395281_1.fast	ftp.sra.ebi.ac.uk/vol1/err/ERR133/081/ERR13395281/ERR13395281.bam
Bac. deg.	Control	43	ERR13395285	SAMEA115854238	ERX12765947	ftp.sra.ebi.ac.uk/vol1/fastq/ERR133/085/ERR13395285/ERR13395285_1.fast	ftp.sra.ebi.ac.uk/vol1/err/ERR133/085/ERR13395285/ERR13395285.bam
Bac. deg.	Control	43	ERR13395288	SAMEA115854241	ERX12765950	ftp.sra.ebi.ac.uk/vol1/fastq/ERR133/088/ERR13395288/ERR13395288_1.fast	ftp.sra.ebi.ac.uk/vol1/err/ERR133/088/ERR13395288/ERR13395288.bam
Bac. deg.	Jelly-OM	43	ERR13395291	SAMEA115854244	ERX12765953	ftp.sra.ebi.ac.uk/vol1/fastq/ERR133/091/ERR13395291/ERR13395291_1.fast	ftp.sra.ebi.ac.uk/vol1/err/ERR133/091/ERR13395291/ERR13395291.bam
Bac. deg.	Jelly-OM	43	ERR13395294	SAMEA115854247	ERX12765956	ftp.sra.ebi.ac.uk/vol1/fastq/ERR133/094/ERR13395294/ERR13395294_1.fast	ftp.sra.ebi.ac.uk/vol1/err/ERR133/094/ERR13395294/ERR13395294.bam
Bac. deg.	Jelly-OM	43	ERR13395297	SAMEA115854250	ERX12765959	ftp.sra.ebi.ac.uk/vol1/fastq/ERR133/097/ERR13395297/ERR13395297_1.fast	ftp.sra.ebi.ac.uk/vol1/err/ERR133/097/ERR13395297/ERR13395297.bam
Bac. deg.	Control	67	ERR13395286	SAMEA115854239	ERX12765948	ftp.sra.ebi.ac.uk/vol1/fastq/ERR133/086/ERR13395286/ERR13395286_1.fast	ftp.sra.ebi.ac.uk/vol1/err/ERR133/086/ERR13395286/ERR13395286.bam
Bac. deg.	Control	67	ERR13395289	SAMEA115854242	ERX12765951	ftp.sra.ebi.ac.uk/vol1/fastq/ERR133/089/ERR13395289/ERR13395289_1.fast	ftp.sra.ebi.ac.uk/vol1/err/ERR133/089/ERR13395289/ERR13395289.bam
Bac. deg.	Control	67	ERR13395292	SAMEA115854245	ERX12765954	ftp.sra.ebi.ac.uk/vol1/fastq/ERR133/092/ERR13395292/ERR13395292_1.fast	ftp.sra.ebi.ac.uk/vol1/err/ERR133/092/ERR13395292/ERR13395292.bam
Bac. deg.	Jelly-OM	67	ERR13395278	SAMEA115854231	ERX12765940	ftp.sra.ebi.ac.uk/vol1/fastq/ERR133/078/ERR13395278/ERR13395278_1.fast	ftp.sra.ebi.ac.uk/vol1/err/ERR133/078/ERR13395278/ERR13395278.bam
Bac. deg.	Jelly-OM	67	ERR13395282	SAMEA115854235	ERX12765944	ftp.sra.ebi.ac.uk/vol1/fastq/ERR133/082/ERR13395282/ERR13395282_1.fast	ftp.sra.ebi.ac.uk/vol1/err/ERR133/082/ERR13395282/ERR13395282.bam
Bac. deg.	Jelly-OM	67	ERR13395300	SAMEA115854253	ERX12765962	ftp.sra.ebi.ac.uk/vol1/fastq/ERR133/000/ERR13395300/ERR13395300_1.fast	ftp.sra.ebi.ac.uk/vol1/err/ERR133/000/ERR13395300/ERR13395300.bam
Phyt. res.	-	0	ERR13395295	SAMEA115854248	ERX12765957	ftp.sra.ebi.ac.uk/vol1/fastq/ERR133/095/ERR13395295/ERR13395295_1.fast	ftp.sra.ebi.ac.uk/vol1/err/ERR133/095/ERR13395295/ERR13395295.bam
Phyt. res.	Control	144	ERR13395283	SAMEA115854236	ERX12765945	ftp.sra.ebi.ac.uk/vol1/fastq/ERR133/083/ERR13395283/ERR13395283_1.fast	ftp.sra.ebi.ac.uk/vol1/err/ERR133/083/ERR13395283/ERR13395283.bam
Phyt. res.	Control	144	ERR13395287	SAMEA115854240	ERX12765949	ftp.sra.ebi.ac.uk/vol1/fastq/ERR133/087/ERR13395287/ERR13395287_1.fast	ftp.sra.ebi.ac.uk/vol1/err/ERR133/087/ERR13395287/ERR13395287.bam
Phyt. res.	Control	144	ERR13395290	SAMEA115854243	ERX12765952	ftp.sra.ebi.ac.uk/vol1/fastq/ERR133/090/ERR13395290/ERR13395290_1.fast	ftp.sra.ebi.ac.uk/vol1/err/ERR133/090/ERR13395290/ERR13395290.bam
Phyt. res.	Jelly-OM	144	ERR13395279	SAMEA115854232	ERX12765941	ftp.sra.ebi.ac.uk/vol1/fastq/ERR133/079/ERR13395279/ERR13395279_1.fast	ftp.sra.ebi.ac.uk/vol1/err/ERR133/079/ERR13395279/ERR13395279.bam
Phyt. res.	Jelly-OM	144	ERR13395298	SAMEA115854251	ERX12765960	ftp.sra.ebi.ac.uk/vol1/fastq/ERR133/098/ERR13395298/ERR13395298_1.fast	ftp.sra.ebi.ac.uk/vol1/err/ERR133/098/ERR13395298/ERR13395298.bam
Phyt. res.	Jelly-OM	144	ERR13395301	SAMEA115854254	ERX12765963	ftp.sra.ebi.ac.uk/vol1/fastq/ERR133/001/ERR13395301/ERR13395301_1.fast	ftp.sra.ebi.ac.uk/vol1/err/ERR133/001/ERR13395301/ERR13395301.bam
In situ	No. Patch	-	ERR13395293	SAMEA115854246	ERX12765955	ftp.sra.ebi.ac.uk/vol1/fastq/ERR133/093/ERR13395293/ERR13395293_1.fast	ftp.sra.ebi.ac.uk/vol1/err/ERR133/093/ERR13395293/ERR13395293.bam
In situ	No. Patch	-	ERR13395296	SAMEA115854249	ERX12765958	ftp.sra.ebi.ac.uk/vol1/fastq/ERR133/096/ERR13395296/ERR13395296_1.fast	ftp.sra.ebi.ac.uk/vol1/err/ERR133/096/ERR13395296/ERR13395296.bam
In situ	No. Patch	-	ERR13395299	SAMEA115854252	ERX12765961	ftp.sra.ebi.ac.uk/vol1/fastq/ERR133/099/ERR13395299/ERR13395299_1.fast	ftp.sra.ebi.ac.uk/vol1/err/ERR133/099/ERR13395299/ERR13395299.bam
In situ	Patch	-	ERR13395280	SAMEA115854233	ERX12765942	ftp.sra.ebi.ac.uk/vol1/fastq/ERR133/080/ERR13395280/ERR13395280_1.fast	ftp.sra.ebi.ac.uk/vol1/err/ERR133/080/ERR13395280/ERR13395280.bam
In situ	Patch	-	ERR13395284	SAMEA115854237	ERX12765946	ftp.sra.ebi.ac.uk/vol1/fastq/ERR133/084/ERR13395284/ERR13395284_1.fast	ftp.sra.ebi.ac.uk/vol1/err/ERR133/084/ERR13395284/ERR13395284.bam
In situ	Patch	-	ERR13395302	SAMEA115854255	ERX12765964	ftp.sra.ebi.ac.uk/vol1/fastq/ERR133/002/ERR13395302/ERR13395302_1.fast	ftp.sra.ebi.ac.uk/vol1/err/ERR133/002/ERR13395302/ERR13395302.bam
