# Supplementary material for: Microbial degradation of jellyfish detritus promotes phytoplankton growth in coastal marine ecosystems
Source: ISME Commun. 2026 Jul 1;6(1):ycag185. doi: 10.1093/ismeco/ycag185 (PMC13452463; doi:10.1093/ismeco/ycag185)
Supplement: Supplementary file 1 [file supplementary_material_ycag185.zip › Figure_S1.pdf]

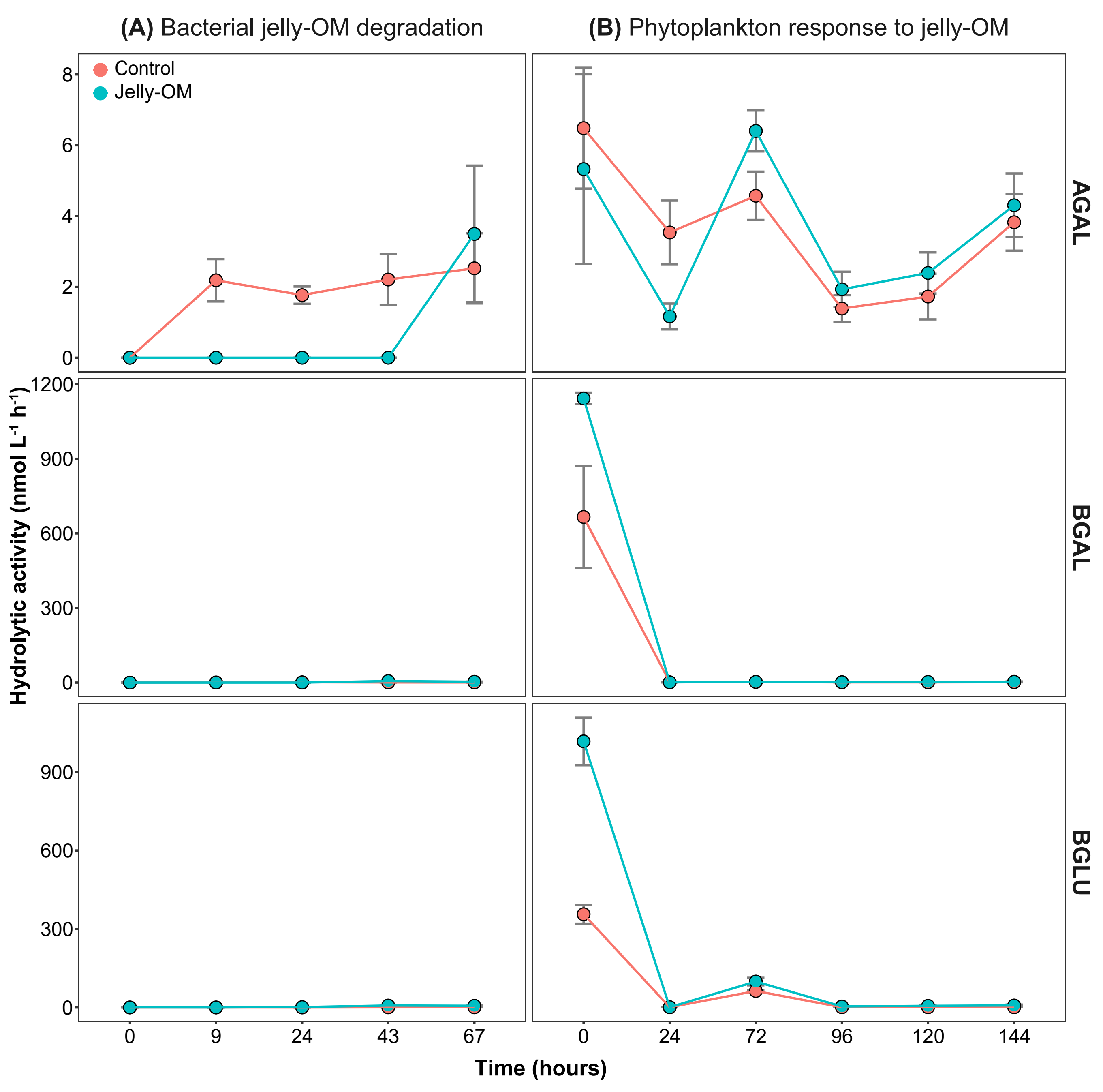

(A) Bacterial jelly-OM degradation
(B) Phytoplankton response to jelly-OM
Control
Jelly-OM
AGAL
BGAL
BGLU
Hydrolytic activity (nmol L-1 h-1)
Time (hours)
0
2
4
6
8
300
600
900
1200
0
9
24
43
67
72
96
120
144
