# Supplementary material for: Microbial degradation of jellyfish detritus promotes phytoplankton growth in coastal marine ecosystems
Source: ISME Commun. 2026 Jul 1;6(1):ycag185. doi: 10.1093/ismeco/ycag185 (PMC13452463; doi:10.1093/ismeco/ycag185)
Supplement: Supplementary file 1 [file supplementary_material_ycag185.zip › Figure_S2.pdf]

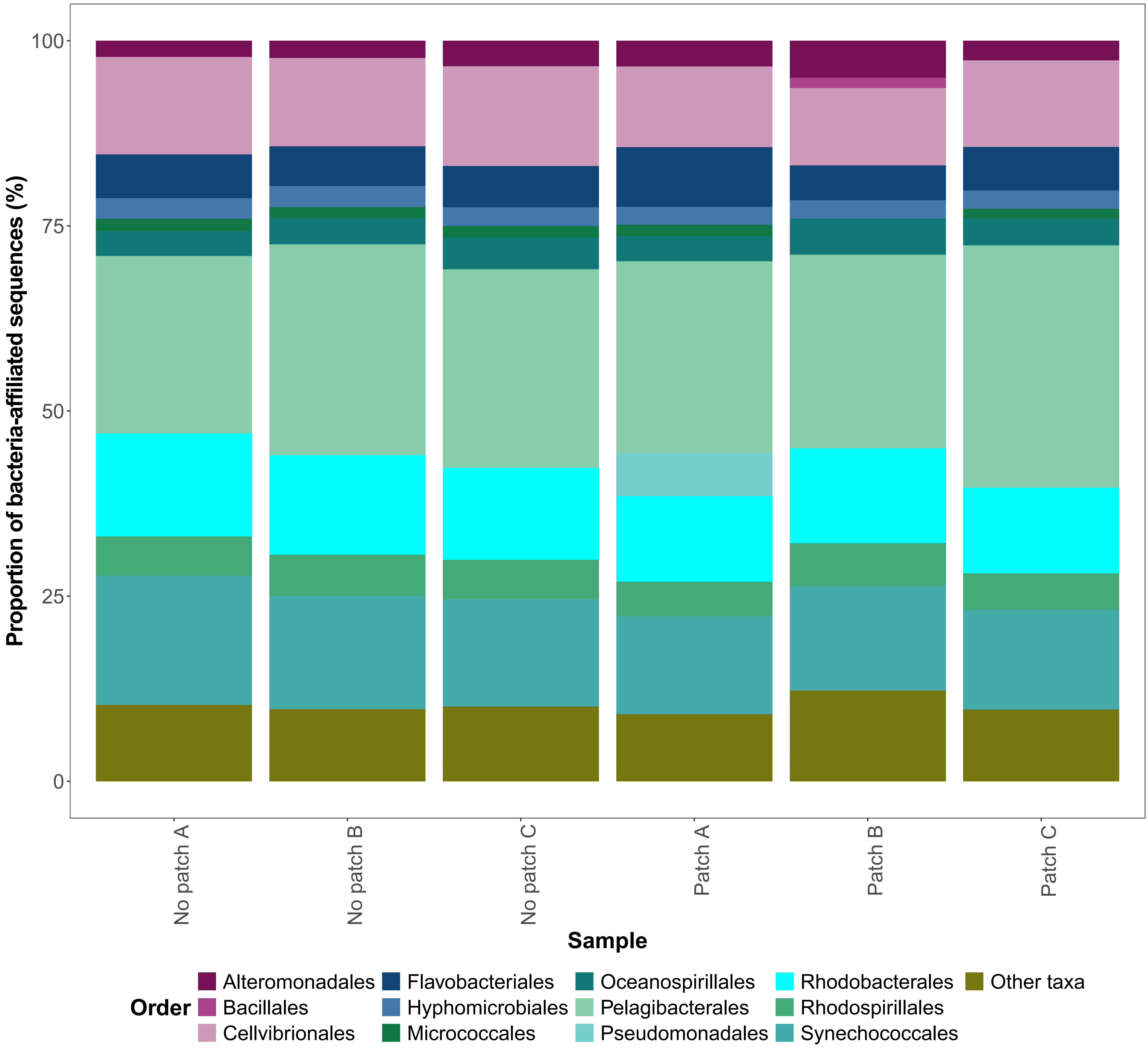

Proportion of bacteria-affiliated sequences (%)
100
75
50
25
0
No patch A
No patch B
No patch C
Patch A
Patch B
Patch C
Sample
Order
Alteromonadales
Bacillales
Cellvibrionales
Flavobacteriales
Hyphomicrobiales
Micrococcales
Oceanospirillales
Pelagibacterales
Pseudomonadales
Rhodobacterales
Rhodospirillales
Synechococcales
Other taxa
