# Supplementary material for: Microbial degradation of jellyfish detritus promotes phytoplankton growth in coastal marine ecosystems
Source: ISME Commun. 2026 Jul 1;6(1):ycag185. doi: 10.1093/ismeco/ycag185 (PMC13452463; doi:10.1093/ismeco/ycag185)
Supplement: Supplementary file 1 [file supplementary_material_ycag185.zip › Supplementary materials_legends.pdf]

Supplementary materials

Supplementary materials S1: Supplementary information for methods section on Prokaryotic abundance and production and Phytoplankton abundance and production

Supplementary Figure 1: Extracellular enzymatic activities measured using fluorogenic substrate analogues derived from 7-amino-4-methyl-coumarin (AMC) and 4-methyl-umbelliferone (MUF). Alpha Galactosidase (AGAL), Beta Galactosidase (BGAL), and Beta Glucosidase (BGLU).

Supplementary Figure 2: Relative abundance of bacteria-affiliated sequences outside (No patch) and inside (patch) *M.leidyi* patch in natural marine microbial communities.

Supplementary Tables:

Table S1: Metadata for metagenomic samples deposited under accession PRJEB77875, including the associated experimental conditions

Table S2 - Concentration of dissolved organic and inorganic nutrients, including chlorophyll a and primary production, bacterial carbon production and abundance throughout two stage microcosm experiment in jelly-OM amended bottles and in control treatments with no jelly-OM addition and in situ.

Table S3 - Bacterial community growth parameters during exponential growth phase in the first stage of our microcosm experiment in jelly-OM amended bottles and in control treatments with no jelly-OM amendment.

Table S4 - Abundance of microbial cells (DAPI), bacterial cells (D+F (DAPI positive cells hybridized with EUB I, II and III probe)), respiring and HPG incorporating microbes (D+H (DAPI and RSG/HPG positive cells)) and respiring and HPG incorporating bacteria (D+F+H (DAPI and RSG/HPG positive cells hybridized with EUB I, II and III probe) at the start (0h) and the end (67, 144h) of each of the stage (Bac. deg, Phyt.res) of our microcosm experiment in jelly-OM amended bottles and in control treatments with no jelly-OM amendment.

Table S5 - Concentration of dissolved free amino acids, dissolved combined amino acids and total hydrolysable dissolved amino acids throughout two stage microcosm experiment in jelly-OM amended bottles and in control treatments with no jelly-OM addition
