# Supplementary material for: Microbial degradation of jellyfish detritus promotes phytoplankton growth in coastal marine ecosystems
Source: ISME Commun. 2026 Jul 1;6(1):ycag185. doi: 10.1093/ismeco/ycag185 (PMC13452463; doi:10.1093/ismeco/ycag185)
Supplement: Supplementary file 1 [file supplementary_material_ycag185.zip › Supplementary materials_methods_S1.pdf]

Supplementary materials for manuscript

'Microbial degradation of jellyfish detritus promotes phytoplankton growth in coastal marine ecosystems'

Tinkara Tinta1*†, Eduard Fadeev2*, Mauro Celussi3, Cecilia Balestra3, Katja Klun1, Patricija Mozetič1 and Gerhard J Herndl2†

Materials and Methods

Prokaryotic abundance and production. Epifluorescence microscopy of DAPI-stained cells was used during the experiment to track microbial dynamics in near real time. For this, two replicates (1.5 mL each) were fixed with 0.2 µm filtered formaldehyde (2% final concentration) and analyzed as previously described [41]. To enumerate abundance of different microbial groups via flow cytometry, two 1.5 mL were fixed with glutaraldehyde (0.5% final concentration). The abundance of prokaryotes and phototrophic pico- and nanoplankton (Synechococcus, picoeukaryotes and nanoeukaryotes) was assessed using a FACSCantoII flow cytometer (Becton Dickinson BioSciences Inc, USA) equipped with an air-cooled argon laser at 488 nm and standard filters setup. After thawing to room temperature, the abundance of phototrophs was determined from unstained samples based on their natural fluorescence (phycoerythrin and chlorophyll *a*; [59]) and analyzed for 3 min at a flow rate of approximately 121 µL min^{-1} using red fluorescence as a trigger. Samples for enumerating total prokaryotic abundance were diluted 10-fold with 0.2 µm filtered Tris-EDTA buffer (1X, Sigma Aldrich), stained with SYBR Green I (1X Final Concentration, Life Technologies), incubated in the dark at room temperature for 10 min according to Marie et al. [60] and analyzed for 2 min at a flow rate of approximately 60 µL min^{-1} using green

fluorescence as a trigger. Data acquisition was performed using FACSDiva software (Becton Dickinson Franklin Lake, USA) and files were analyzed using FCS Express 7 Research (7.18.0021, DeNovo Software, Glendale, USA).

Phytoplankton abundance and production. Composition and abundance of phytoplankton were analyzed in formaldehyde-fixed (2% final concentration) samples (500 mL) according to the Utermöhl method [66]. Micro- and nanophytoplankton were identified down to the smallest possible taxon and counted in subsamples of 50 or 25 mL, which were allowed to sediment overnight. Depending on the abundance in the subsamples, 50 - 100 fields of the bottom sedimentation chamber were examined at 400x magnification on an inverted microscope (Zeiss Axio Observer Z1). Chlorophyll *a* (Chl *a*) concentrations as a proxy for phytoplankton biomass, corrected for phaeopigments, were determined fluorometrically in 90% acetone extracts on a Turner Trilogy fluorometer [67]. Primary production (PP) of phytoplankton was measured using the ^{14}C method [68]. Seawater (75 ml) from each treatment was placed in polycarbonate bottles (2 light and 1 dark), spiked with 185 kBq $NaH^{14}CO_3$ and incubated for 4 h under controlled in vitro conditions (at ambient seawater temperature and an irradiance of approx. 170 µmol photons m^{-2} s^{-1}). After incubation, subsamples were filtered onto 25 mm GF/F filters (~0.8 µm pore size; Whatman), acidified overnight with 250 µL 0.1 M HCl to remove inorganic carbon. After the addition of 5 mL Ultima Gold scintillation cocktail, the activity of the samples was determined on a TriCarb 3100TR liquid scintillator. The assimilation of carbon was calculated as described by

Gargas [69], considering a 5% isotope discrimination, the activity of the added ^{14}C and the total CO_2 concentration calculated from temperature, salinity and pH.
